# Tissue amino acid profiles are characteristic of tumor type, malignant phenotype, and tumor progression in pancreatic tumors

**DOI:** 10.1038/s41598-019-46404-4

**Published:** 2019-07-08

**Authors:** Nobuyoshi Hiraoka, Sakino Toue, Chisato Okamoto, Shinya Kikuchi, Yoshinori Ino, Rie Yamazaki-Itoh, Minoru Esaki, Satoshi Nara, Yoji Kishi, Akira Imaizumi, Nobukazu Ono, Kazuaki Shimada

**Affiliations:** 1 Division of Molecular Pathology, National Cancer Center Research Institute, Tokyo, Japan; 2 Division of Pathology and Clinical Laboratories, National Cancer Center Hospital, Tokyo, Japan; 3 Hepato-Biliary and Pancreatic Surgery Division, National Cancer Center Hospital, Tokyo, Japan; 4 Research Institute for Bioscience Products and Fine Chemicals, Ajinomoto Co., Inc, Kanagawa, Japan; 5 Institute for Innovation, Ajinomoto Co., Inc, Kanagawa, Japan

**Keywords:** Cancer metabolism, Pancreatic cancer, Diagnostic markers, Prognostic markers

## Abstract

Tissue amino acid profiles depend on the cell types and extracellular components that constitute the tissue, and their functions and activities. We aimed to characterize the tissue amino acid profiles in several types of pancreatic tumors and lesions. We examined tissue amino acid profiles in 311 patients with pancreatic tumors or lesions. We used newly developed LC-MS/MS methods to obtain the profiles, which were compared with clinicopathological data. Each tumor or lesion presented a characteristic tissue amino acid profile. Certain amino acids were markedly altered during the multistep pancreatic carcinogenesis and pancreatic ductal adenocarcinoma (PDAC) progression. A tissue amino acid index (TAAI) was developed based on the amino acids that were notably changed during both carcinogenesis and cancer progression. Univariate and multivariate survival analyses revealed that PDAC patients with a high TAAI exhibited a significantly shorter survival rate, and these findings were validated using a second cohort. We suggest that tissue amino acid profiles are characteristic for normal tissue type, tumor histological type, and pathological lesion, and are representative of the cancer grade or progression stage in multistep carcinogenesis and of malignant characteristics. The TAAI could serve as an independent prognosticator for patients with PDAC.

## Introduction

Cellular metabolism varies according to distinct physiological and pathological states; thus, tissue metabolite profiles represent the sum of the functional state and biological activity of the cells that constitute the tissues. As the unique physiology of cancer creates a hostile and nutrient-poor microenvironment, cancer cells exhibit biochemical and metabolic adaptations^1^ for surviving and proliferating in non-native settings under conditions of nutrient and oxygen deprivation, and immune-cell attack^2^. Cancer cells shift to anaerobic glycolysis, and accelerate the TCA anaplerosis pathway to produce sufficient energy from Gln and synthesize adequate proteins and nucleotides for proliferating in the hostile microenvironment^3,4^. Gln is also used in redox balance in certain cancers^5,6^. Moreover, when cancers are hypovascular and in a nutrient-deprived state^7,8^, as in the case of pancreatic cancer, cancer cells seek out alternative sources of nutrients: cancer cells are observed to recycle intracellular nutrients^9,10^, access nontraditional extracellular nutrients by scavenging the extracellular space^7,8,11,12^, and engage in metabolic crosstalk with nonmalignant stromal cells, such as cancer-associated fibroblasts, in the tumor microenvironment^13–16^.

In most previous studies in which amino acid concentrations were measured in tissues of surgical specimens, target tissue regions were selected through macroscopic observation. However, as cancer tissues are heterogeneous, the selected cancer tissues occasionally contain surrounding non-cancerous tissues. It is a challenging task to collect cancer tissues from tumors that lack a clear border (invasive tumors), such as pancreatic cancer. Therefore, it is crucial to obtain the detailed information of tissues and their contents while investigating tissue amino acid profiles. Recently, we developed a new technique for measuring with high sensitivity and reproducibility, amino acids extracted from thin-sliced frozen tissues embedded in an optical cutting temperature (OCT) compound^17^. For histological examination, standard frozen tissue sections can be generated from frozen tissues embedded in OCT compound and tissue amino acid profiles can be obtained along with histological information.

Pancreatic ductal adenocarcinoma (PDAC), the most lethal type of cancer, features a 5-year survival rate of only 8%^18^. PDAC exhibits aggressive growth and early metastatic dissemination; moreover, due to the absence of clinically informative early symptoms and diagnostic biomarkers, most patients do not receive timely treatment by curative surgical resection. Thus, the development of biomarkers for detecting PDAC in the early or premalignant stage, and selection of patient subsets for treatment will facilitate reduction of mortality in patients with PDAC. Recently, we reported a multivariate index for PDAC detection based on the plasma-free amino acid profile^19^, although the clinicopathological impact of the tissue amino acid profile in PDAC has not yet been elucidated. Besides PDAC, several other epithelial tumors develop in the pancreatic tissues, including acinar cell carcinoma (ACC), neuroendocrine tumor (NET), and solid-pseudopapillary neoplasm (SPN)^20^. These pancreatic tumors are classified mostly based on the tumor-cell phenotype corresponding to a component of the normal pancreatic tissue, such as pancreatic-duct-covering epithelial cells, acinar cells, or islet cells, with the exception of SPN with incompletely identified normal counterpart^20^. As noted above, metabolism of cancer tissues differs from the corresponding non-cancerous tissues, with the most common metabolic activity of cancer cells being glycolysis^21^. Certain metabolic profiles have been characterized as tissue-specific or cancer-specific by comparing the profiles among liver, breast, and pancreatic cancers^22^. However, we still do not understand whether tissue amino acid profiles (1) differ according to the tumor types that develop within the same tissue, (2) change during multistep carcinogenesis, and (3) are associated with the clinical behavior of a tumor.

Here, we examined tissue amino acid profiles in 311 patients with pancreatic tumors or lesions. This is the first report showing that tissue amino acid profiles are a characteristic feature of the tumor histological types or lesions that develop in the pancreas. We also show that tissue amino acid profiles change according to the tumor progression stage during multistep pancreatic carcinogenesis, and that tissue amino acid index (TAAI), generated based on selected amino acids closely correlated with PDAC development and progression, is a prognosticator of patient outcomes. Our findings suggest that tissue amino acid profiles can provide information for predicting patient outcomes.

## Results and Discussion

### Tissue amino acid profiles of normal and PDAC tissues

**Figure 1:**
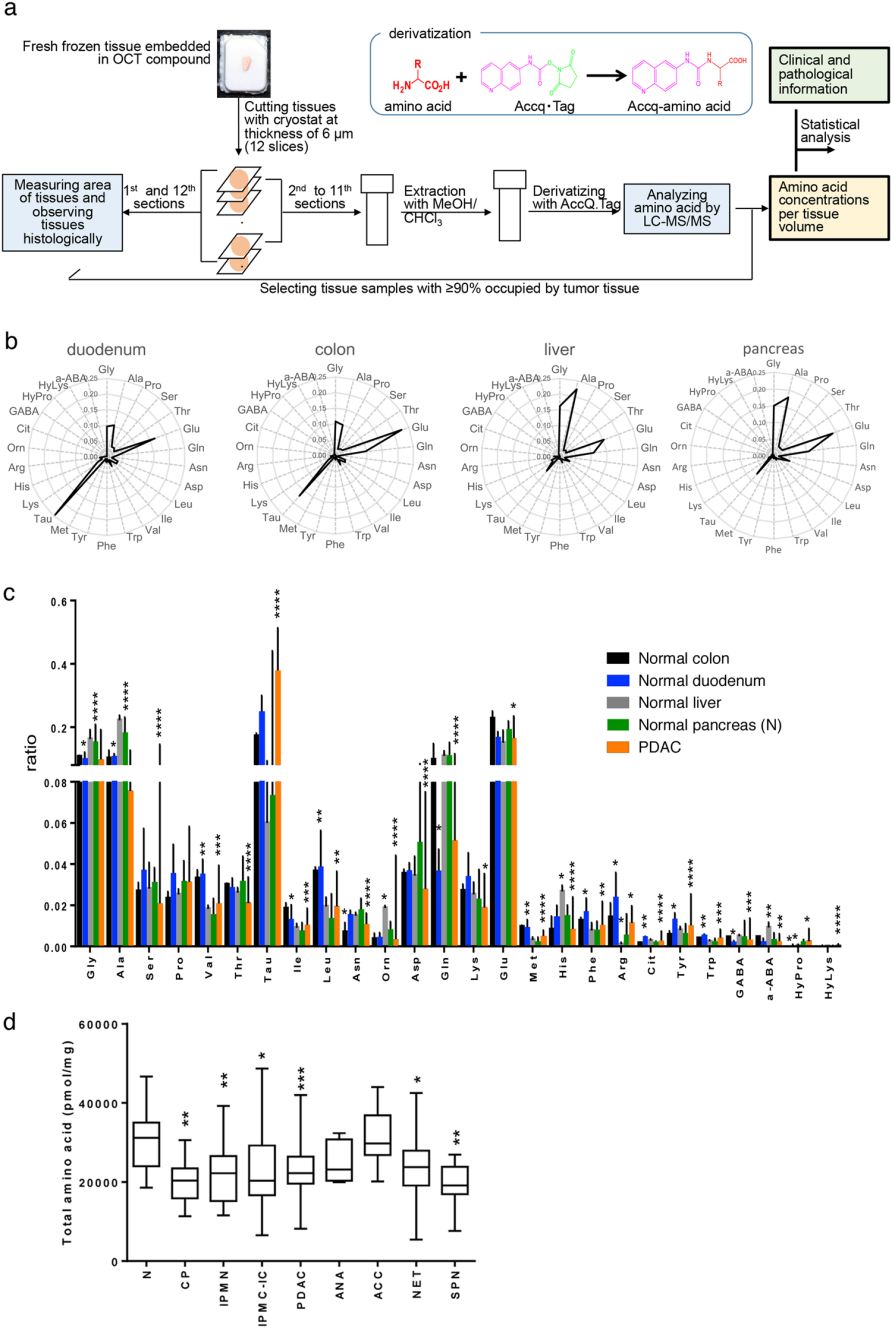
Characteristics of amino acid profile in normal and pancreatic cancer tissues. (**a**) Scheme of sample analysis. (**b**) Radar charts of amino acid concentration ratios in normal tissues, including liver, duodenum, colon, and pancreas. Data represent median of amino acid concentration ratios in each tissue type. (**c**) Ratio of amino acid concentrations in normal tissues and PDAC. Values are median + quartile range. *, **, ***, ****: *P* < 0.05, 0.01, 0.001, 0.0001 (Steel comparison test, versus normal pancreas). (**d**) Differences in amino acid concentrations between normal and diseased pancreatic tissues. *, **, ***, ****: *P* < 0.05, 0.01, 0.001.

Surgically resected tissue samples were immediately frozen and embedded in OCT compound (Fig. 1a), and the amino acids were extracted from cryostat sections. The samples were maintained under cold conditions during extraction, thus minimizing sample degradation. Serial cryostat sections were analyzed histologically and the data was obtained along with the amino acid profiles. We used frozen tissues in which tumor tissue/lesion occupied >90% of the total tissue area in cryostat sections (240/323 cases, Table 1). A newly developed LC-MS/MS method^17^ was utilized to measure 26 amino acids quantitatively (Supplementary Table S1).

**Table 1:** List of patients enrolled in this study.

Disease	number of cases
**Ductal adenocarcinoma (PDAC)**	
common type	130
anaplastic carcinoma	6
**Intraductal papillary-mucinous neoplasm (IPMN)**	
low- and intermediate-grade dysplasia (IPMA)	13^a^
high-grade dysplasia (IPMC)	13^b^
associated with invasive carcinoma (IPMC-IC)	18^c^
**Acinar cell carcinoma (ACC)**	10^d^
**Neuroendocrine tumor (NET)**	25^e^
**Solid-pseudopapillary neoplasm (SPN)**	10
**Chronic pancreatitis (CP)**	
non-specific	10
Lymphoplasmacytic sclerosing pancreatitis	5
**Normal tissue**	
pancreas (N)	18
liver	4
duodenum	4
stomach	1
colon	3

^a^Contains gastric type (n = 9) and intestinal type (n = 3).

^b^Contains gastric (n = 4), intestinal (n = 3), pancreatobiliary (n = 3), and oncocytic (n = 3) types.

^c^Contais intestinal (n = 4) and pancreatobiliary (n = 14) types.

^d^Contains pancreatoblastoma (n = 2) and mixed acinar-neuroendocrine carcinoma (n = 2).

^e^Contains NETG1 (n = 2), NETG2 (n = 20), and NETG3 (n = 3).

**Figure 2:**
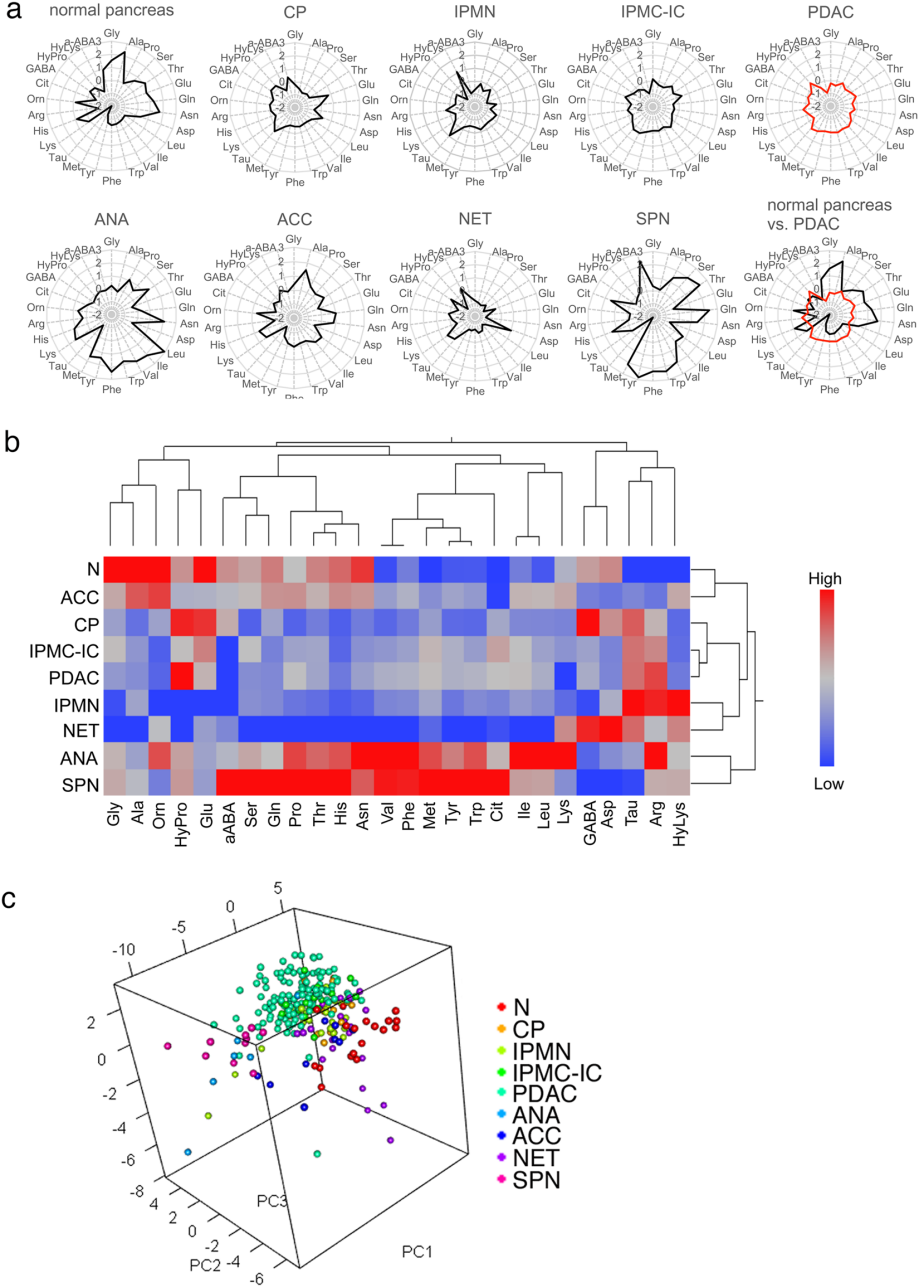
Amino acid profiles of normal and diseased pancreatic tissues. (**a**) Radar charts of standardized amino acid profiles of normal and diseased pancreatic tissues from chronic pancreatitis (CP), intraductal papillary-mucinous neoplasm (IPMN), IPMN associated with invasive carcinoma (IPMC-IC), pancreatic ductal adenocarcinoma (PDAC), anaplastic carcinoma (ANA), acinar cell carcinoma (ACC), neuroendocrine tumor (NET) and solid-pseudopapillary neoplasm (SPN). Data shown are medians of z-scores calculated from the values of amino acid concentration ratios in each tissue type. (**b**) Hierarchical cluster analysis of median amino acid concentration ratios in each tissue type. (**c**) Three-dimensional scatter plots of PCA scores for each tissue.

Tissue amino acid profiles differed among normal colon, duodenum, liver, and pancreas (Fig. 1b), although the profiles of colon and duodenum, as well as that of liver and pancreas were similar. It is suggested that different tissues with similar organ structures and tissue components have similar amino acid profiles. Adenocarcinomas of stomach or colon featured tissue amino acid profiles that exhibited upregulation of most amino acids relative to normal tissue counterparts^21^. By contrast, relative to the normal pancreas (N) profile, PDAC tissue amino acid concentrations showed significant downregulation of many amino acids, although some were significantly upregulated (Figs 1c and 2a). In addition, total amino acid concentration in N was higher than that in all types of pancreatic tumors, except ACC and ANA (Fig. 1d).

### Tissue amino acid profiles are characteristic of histological type of pancreatic tumor or lesion

Tissue amino acid profiles of several pancreatic tumors and lesions appeared different in radar charts (Fig. 2a). Moreover, each type of pancreatic tumor/lesion and N were separated by hierarchical clustering performed using both the median values of cases presenting the same histological types (Fig. 2b), and individual values of cases (data not shown). Interestingly, tissue amino acid profiles were similar between ACC and N, and between PDAC and CP, irrespective of the clustering made between the normal tissue and neoplasm. It is assumed that the clustering was affected by the common major tissue component; acinar cells constitute the majority of the cellular components of the normal pancreatic parenchyma, whereas for CP and PDAC, fibrous stroma forms the main background tissue.

Principal component analysis (PCA) also revealed that tumor or tissue types were classified according to their distinct patterns of constituent amino acids (Fig. 2c and Supplementary Fig. S1). These results suggest that distinct tissue types present characteristic amino acid profiles.

### Tissue components are directly related to the amino acid concentration

**Figure 3:**
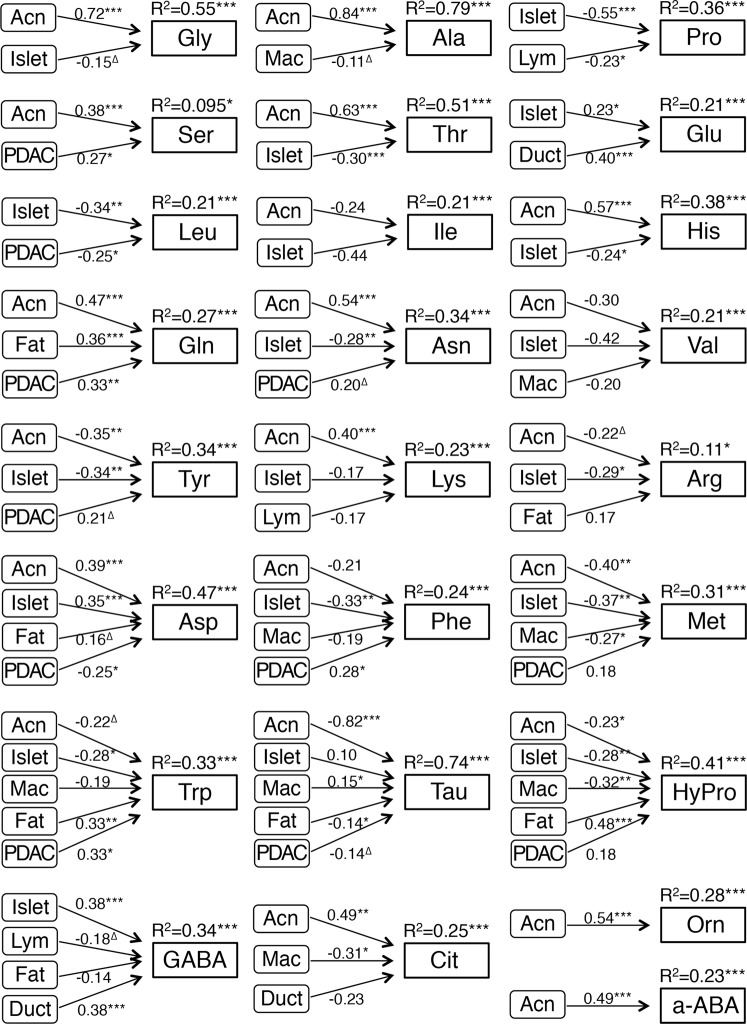
The direct relation between tissue amino acid concentration and tissue components. Path analysis (multiple regression analysis) is performed using N (n = 7), CP (n = 10), and PDAC (n = 53). The direct relationship between tissue components and amino acid concentration is represented by means of low diagram. R^2^ (coefficient of determination) is adjusted r-squared that the model explains all the variability of the response data around its mean. Path coefficients (standard partial regression coefficients) estimate the strength of the relationship between two variables. Tissue occupancy of each tissue component [Acn: Bcl-10 (331.3)^+^ acinar cells, Islet: Chromogranin A (CGA)^+^ islet cells, Duct: EMA^+^ or Cytokeratin (CK, AE1/AE3)^+^/Bcl-10^−^/CGA^−^ ductal epithelial cells in non-cancerous tissue, Mac: CD45^+^/CD68^+^ macrophages, Lym: CD45^+^/CD68^−^ lymphocytes, PDAC: EMA^+^ or CK^+^/Bcl-10^−^/CGA^−^ cancer cells, Fib: aniline blue^+^ area, Fat: SudanIII^+^ fat cells] is counted as the ratio of its area within the total area. Fib is omitted in calculation for inhibiting multicollinearity. ^∆^, *, **, ***: *P* < 0.10, 0.05, 0.01, 0.001. For example, the tissue concentration of Thr is directly and significantly correlated with the volume of Acn positively and with the volume of Islet negatively. Their standard partial regression coefficients are 0.63 and −0.30, respectively. The R^2^ (coefficient of determination) is 0.51, meaning 51% of the tissue Thr concentration is affected by these factors, Acn and Islet.

The tissues are composed of several components (i.e. fibrous tissue, fat tissue, acinar cells, islet cells, cancer cells, macrophages, and immune cells). It is possible that these tissue components have their own specific amino acid profiles. In this scenario, tissue amino acid profiles should correlate with the tissue occupancy rate of each component. Our path analysis (multivariate regression analysis) revealed that tissue occupancy rate of each tissue component was directly related to the concentration of almost all amino acids measured (Fig. 3). These relations were of various degree and strength. More than 30% of the concentration of Gly (55%), Ala (79%), Pro (36%), Thr (51%), His (38%), Asn (34%), Tyr (34%), Asp (47%), Trp (33%), Tau (74%), HyPro (41%), and gamma-amino-n-butyric acid (GABA) (34%) could be explained by the occupancy rate of tissue components. Furthermore, more than 50% of the concentration of Gly (79%), Ala (85%), Pro (64%), Ser (56%), Thr (71%), His (67%), Gln (71%), Asn (65%), Trp (59%), Tau (97%), HyPro (84%), or Orn (71%) were related, when we analyzed the relationship only in the normal and chronic inflamed tissues (Supplementary Fig. S2). These amino acids were rich in N compared to PDAC. In contrast, concentrations of Leu, Tyr, Phe, and Met were directly related to PDAC cell component together with non-cancerous components, although their contributions were not so high (Fig. 3). These amino acids have been reported to be facilitated in uptake and usage in PDAC cells and are more abundant in PDAC compared to N. These results suggest that tissue components, especially in acinar cells and islet cells, are major factors that explain tissue amino acid concentrations in non-cancerous and cancerous pancreatic tissues. It is probably due to a relatively constant cellular and metabolic activity in each tissue component of non-cancerous tissues. In contrast, PDAC tissues are usually more heterogeneous, as both the PDAC cells and their microenvironment have heterogeneous characters. Thereby each tissue component is metabolically heterogeneous among PDAC cases, and the volume ratios of tissue components might not be strong factors in PDAC.

### Summary of the characteristic tissue amino acid profiles of each tumor

We summarize the characteristic tissue amino acid profile of each tumor.

#### PDAC

Tissue amino acid profiles in PDAC differed from those in N, but were similar to those in CP. As compared with N, PDAC showed a significant reduction in the concentrations of Gly, Ala, Ser, Thr, Asn, Asp, Orn, Gln, His, GABA, and alpha-amino-n-butyric acid (a-ABA), and a significant increase in the concentrations of Val, Tau, Ile, Leu, Met, Phe, Cit, Tyr, Trp, and HyLys. Tissue amino acid concentrations of both PDAC and CP were generally lower than that of N, and for both tissues, normal components are replaced by a fibrous stroma, which forms the main background tissue. In addition, the differences in the amino acid profiles between PDAC and CP were present. For example, the concentration of Pro, Ile, Phe, and Tyr was higher in PDAC than in CP. Most of these amino acids are characteristics of the PDAC cells (Fig. 3), and are involved in cell proliferation and angiogenesis. No significant correlation was found between the amino acid profiles and other clinicopathological variables, such as tumor size and tumor location.

The difference in profiles between PDAC and N is similar to that of hepatocellular carcinoma (HCC) and non-cancerous liver tissue^23^. On the other hand, the difference in profiles between PDAC and N was not similar to that of gastric cancer and non-cancerous stomach tissue^21^, or the colon cancer and non-cancerous colon tissue^21,24^.

Most previous studies evaluated metabolite profiles in both normal and tumor tissues^21,24–26^. However, the metabolite profiles of the cancer tissues could not be directly compared as they were not standardized and were measured under different conditions. Budhu *et al*. performed metabolomic profiling for paired tumor and nontumor liver, breast, and pancreatic tissues, and showed that the metabolites were primarily unique to each tissue and cancer type^22^.

#### ANA

In contrast to the common type PDAC (grades 1–3), ANA (PDAC grade 4), which is listed as a PDAC variant in WHO tumor histological classification^20^, frequently exhibits rapid growth together with medullary features, but with little fibrous stroma. ANA amino acid profiles reflected the metabolism of relatively pure cancer cells, where the concentration of Leu, Ile, Val, Trp, Phe, Tyr, and Met were higher than those in N. These profiles were similar to those in PDAC, although the relative concentration of each amino acid in ANA was considerably higher than in PDAC. PDAC tissue typically contains abundant fibrous stroma similar to CP. Accordingly, PDAC amino acid profiles were similar to a mixture of CP and ANA profiles.

Intriguingly, ANA and SPN featured similar profiles even though their clinical and biological behaviors differ markedly: ANA is highly aggressive, whereas SPN is indolent. ANA and SPN share the histological characteristic of medullary tumor growth with frequent bleeding and necrosis or degeneration. These two tumors showed similar amino acid profiles with the exception of SPN that exhibits a higher a-ABA than in ANA.

#### ACC

Amino acid profiles of ACC and N were quite similar with only two significantly different amino acids concentrations, GABA and HyLys; this might be expected because ACC presents an acinar-cell phenotype, and N is composed of mostly acinar cells ( >80%). Conversely, GABA and Asp were lower in ACC than in N. GABA is synthesized by glutamic acid decarboxylase in neurons and β-cells in Langerhans islets^27^. Asp is present in the central nervous system and in various neuroendocrine cells, including islet cells, most alpha-cells, and a subpopulation of F-cells^28,29^. Concentrations of Asp and GABA were lower in ACC than in N probably because Langerhans islets are present in N.

#### NET

NET presents the phenotype of neuroendocrine cells that are found in Langerhans islets as a major component. Thus, relative to N, NET typically showed higher Asp, and occasionally higher GABA levels. However, in NET, the concentrations of Gly, Ala, Pro, Ser, Thr, Gln, Asn, His, and HyPro were lower than those in other tissue types examined here, and the concentrations of Leu, Ile, Val, Trp, Phe, Tyr, Met, and Cit were lower than those in other tumors examined.

#### SPN

SPN presented a unique profile: Pro, Ser, Thr, Gln, Asn, Val, Trp, Phe, Tyr, Met, His, Cit, and a-ABA levels were higher than in other cases; most of these except Cit and a-ABA were at similar level to that in ANA. By contrast, Asp and Tau were low in SPN. Pancreatic a-ABA level in SPN was higher than that in all other tumors, CP, and N. Similarly, a-ABA does not participate in protein synthesis, and is mainly considered as the product of the metabolism of Met, Thr, Ser, and Gly, derived from alpha-ketobutyrate through transamination^30–33^. The alternative fate of alpha-ketobutyrate is decarboxylation, and formation of propionyl-CoA, succinyl-CoA, and an entry into the Krebs cycle. An increase in plasma a-ABA is considered a non-specific marker of liver dysfunction, malnutrition, sepsis, increased protein catabolism, or a combination of these changes^30–34^ Impaired entry of alpha-ketobutyrate into the Krebs cycle is suggested to contribute towards increasing a-ABA level in these scenarios. Based on these results, and the observation that SPN typically shows indolent growth and can be readily degenerated, we speculate that the metabolic pathway of alpha-ketobutyrate entry into the Krebs cycle is hindered in SPN.

### Certain tissue amino acids are characteristically altered during progression of multistep carcinogenesis

**Figure 4:**
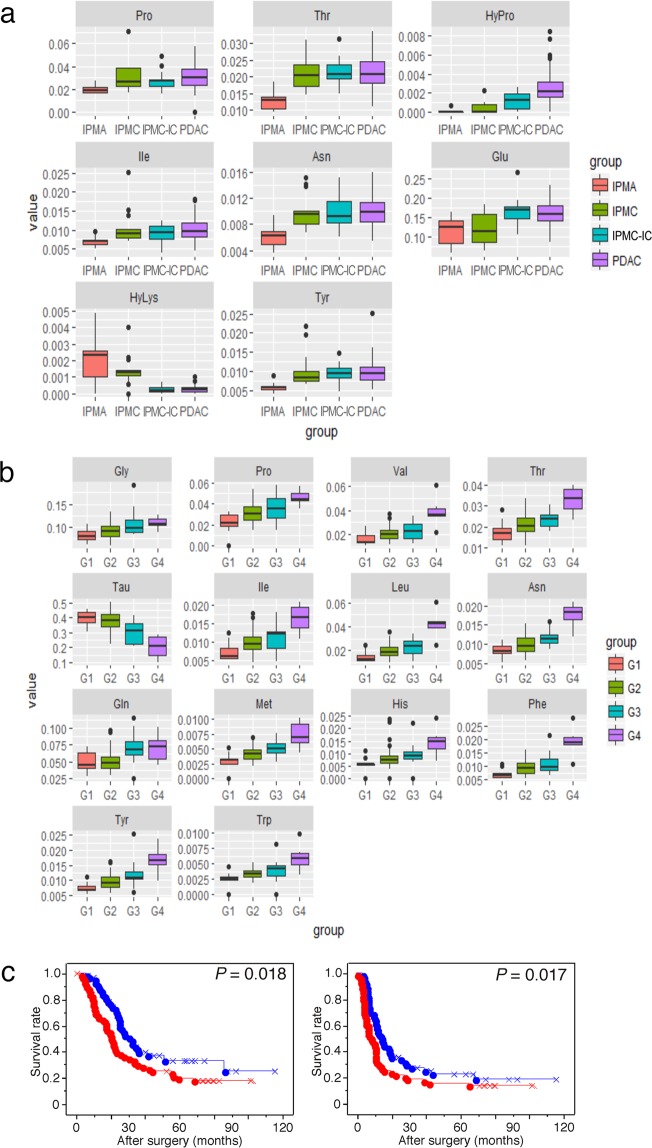
Changes in tissue amino acid concentrations during progression of pancreatic carcinogenesis. (**a**,**b**) Amino acids altered during the progression of multistep pancreatic carcinogenesis in (**a**), intraductal papillary-mucinous neoplasm (IPMN) with low grade dysplasia, alternatively intraductal papillary-mucinous adenoma (IPMA), IPMN with high grade dysplasia, alternatively intraductal papillary-mucinous carcinoma (IPMC), IPMN associated with invasive carcinoma (IPMC-IC), and pancreatic ductal adenocarcinoma (PDAC), and PDAC of various grades in (**b**). Box plots of amino acid concentration ratios are shown. (**c**) Kaplan-Meier survival curves showing comparison of overall survival (left panel) and disease-free survival (right panel) between high (red) and low (blue) of tissue amino acid index (TAAI) groups in cohort 1. *P* values were obtained from log-rank tests. The “×” and “+” represent censoring and failure, respectively.

Next, we determined whether tissue amino acid profiles change during the progression of pancreatic multistep carcinogenesis. PDAC has three premalignant pathways^20^. In the major pathway, pancreatic intraepithelial neoplasia (PanIN) are microscopic lesions and MCN is extremely rare neoplasm, we collected and analyzed IPMN. IPMNs are macroscopic lesions that progress from low-grade to intermediate-grade and then high-grade dysplasia corresponding to *carcinoma in situ*. The concentrations of Pro, Thr, HyPro, Ile, Asn, Glu, and Tyr increased significantly during IPMN progression (Fig. 4a). As HyPro is mainly provided by collagen degradation, and pancreatic invasive cancers have an abundant fibrous stroma, HyPro concentration might be associated with cancer-infiltrating stromal volume. Both Kras and p53 proteins allow the upregulation of glutaminase, which metabolizes Gln to Glu^6^. In IPMN progression, besides *TP*5*3*, *GNAS* is predominantly mutated; however, the *KRAS* mutations have also been observed^35,36^. Thus, the accumulation of *KRAS* and *TP53* mutations might lead to increased Glu during multistep carcinogenesis.

The concentrations of Gly, Pro, Val, Thr, Ile, Leu, Asn, Gln, Met, His, Phe, Tyr, and Trp increased significantly during pancreatic cancer progression, from well-differentiated (G1), to moderately differentiated (G2), then to poorly differentiated adenocarcinoma (G3), and finally to anaplastic carcinoma (G4) (Fig. 4b). Tau concentration decreased significantly during PDAC progression. Given that Leu, Ile, Val, Trp, Phe, Tyr, Met, and His are taken up through LAT1 transporter, LAT1 expression might increase with an increase in PDAC grade. High expression of LAT1 is associated with poor outcome in patients with PDAC^37^. Tau plays diverse biological roles, and is upregulated in several cancers^38,39^. Our results suggest that Tau might be implicated in apoptosis and/or osmotic regulation in CP and several tumor tissues, but is not correlated with malignant phenotype in pancreatic tumors.

### Prognostic significance of TAAI

Our results suggested that the amino acids involved in the progression of multistep carcinogenesis exhibit an association with PDAC malignant behavior. Thus, we attempted the prediction of patient outcome based on the tissue amino acids profiles of 5 amino acids (Pro, Thr, Ile, Asn, and Tyr) whose concentrations were commonly and significantly altered during IPMN-associated pancreatic carcinogenesis and PDAC progression (Fig. 4a,b). To avoid the potential for multicollinearity and/or overfitting, a variable selection process was performed to minimize the Akaike Information Criterion (AIC) of the model. Finally, three tissue amino acid profiles (Pro, Thr, and Tyr) were chosen as the explanatory variables of the TAAI. We generated ROC curves to evaluate TAAI performance as a prognostic biomarker for the survival of PDAC patients. Corresponding AUC and cutoff levels were calculated, which yielded AUC = 0.63 (Supplementary Fig. S1C). When Cohort 1 PDAC patients were dichotomized into high and low TAAI groups, the calculated median survival time was 16.5 and 25.1 months, respectively, and the 1-, 2-, and 5-year survival rates were 66.8 ± 6.1% and 93.2 ± 3.3%, 40.9 ± 6.6% and 67.1 ± 6.4%, and 17.3 ± 5.3% and 33.6 ± 7.1%, respectively.

Univariate survival analysis revealed an association between higher TAAI and shorter OS (*P* = 0.018) and DFS (*P* = 0.017) (Fig. 4c). Multivariate Cox regression-analysis results showed that TAAI (*P* = 0.002; HR = 2.239; 95% CI: 1.328–3.773), age, lymphatic invasion, and venous invasion were independent predictors of OS, and that age, pathologic node status, pathologic metastasis status, histological grade, lymphatic invasion (Table 2), and venous invasion were independent predictors of DFS (Table 3).

**Table 2:** Univariate and multivariate analysis of prognostic factors associated with overall survival in patients with PDAC (cohort 1) (n = 130).

Variables	Univariate analysis		Multivariate analysis	
	HR (95% CI)	*P* value	HR (95% CI)	*P* value
Age (<60/≥60 years)	2.205 (1.409–3.452)	**0.0005**	1.845 (1.129–3.015)	**0.015**
Gender (male/female)	0.791 (0.507–1.233)	0.300		
Pathologic tumor status (T2 + T3/T1)	3.281 (0.806–13.358)	0.097		
Pathologic node status (N1 + N2/N0)	2.425 (1.359–4.330)	**0.003**		
Pathologic metastasis status (M1/M0)	2.373 (1.247–4.515)	**0.009**		
Histological grade (G2 + G3/G1)	2.169 (0.794–5.925)	0.131		
Tumor margin status (positive/negative)	2.322 (1.481–3.640)	**0.0002**		
Lymphatic invasion (2, 3/0, 1)	2.713 (1.597–4.609)	**0.0002**	2.067 (1.197–3.571)	**0.009**
Venous invasion (2, 3/0, 1)	2.146 (1.204–3.828)	**0.010**	1.975 (1.070–3.647)	**0.030**
Intrapancreatic neural invasion (2, 3/0, 1)	2.748 (1.665–4.537)	**<0.0001**	1.784 (1.041–3.057)	**0.035**
Nerve plexus invasion (positive/negative)	1.979 (1.172–3.343)	**0.011**		
Chemotherapy (negative/positive)	1.058 (0.670–1.669)	0.810		
Tissue amino acid index (high/low)	1.689 (1.089–2.622)	**0.019**	1.832 (1.169–2.869)	**0.008**

**Table 3:** Univariate and multivariate analysis of prognostic factors associated with disease-free survival in patients with PDAC (cohort 1) (n = 130).

Variables	Univariate analysis		Multivariate analysis	
	HR (95% CI)	*P* value	HR (95% CI)	*P* value
Age (<60/≥60 years)	1.944 (1.272–2.972)	**0.002**	2.658 (1.677–4.213)	**<0.0001**
Gender (male/female)	0.737 (0.494–1.100)	0.135		
Pathologic tumor status (T2 + T3/T1)	2.132 (0.783–5.805)	0.139		
Pathologic node status (N1 + N2/N0)	2.978 (1.731–5.124)	**<0.0001**	2.341 (1.338–4.097)	**0.003**
Pathologic metastasis status (M1/M0)	2.739 (1.589–4.722)	**0.0003**	2.292 (1.300–4.042)	**0.004**
Histological grade (G2 + G3/G1)	2.651 (1.077–6.524)	**0.034**	2.764 (1.085–7.041)	**0.033**
Tumor margin status (positive/negative)	1.821 (1.207–2.750)	**0.004**		
Lymphatic invasion (2, 3/0, 1)	2.451 (1.532–3.922)	**0.0002**		
Venous invasion (2, 3/0, 1)	2.575 (1.480–4.482)	**0.0008**	2.777 (1.555–4.957)	**0.0006**
Intrapancreatic neural invasion (2, 3/0, 1)	2.733 (1.758–4.248)	**<0.0001**		
Nerve plexus invasion (positive/negative)	1.788 (1.135–2.815)	**0.012**		
Chemotherapy (negative/positive)	1.062 (0.702–1.609)	0.775		
Tissue amino acid index (high/low)	1.607 (1.085–2.380)	**0.018**	1.682 (1.126–2.511)	**0.011**

To confirm the prognostic impact of TAAI, we performed survival analysis by using Cohort 2, where we prospectively collected samples. Univariate and multivariate analyses confirmed that both OS and DFS were significantly shorter for high-TAAI-group patients than for low-TAAI-group patients (Supplementary Fig. S1D and Supplementary Tables S2 and S3). Thus, TAAI is an indicator of poor prognosis for patients with PDAC.

We also examined the correlation of TAAI with clinicopathological characteristics of patients with PDAC (Table 4 and Supplementary Table S4). There was a tendency of a higher TAAI being found in PDACs with a higher histological grade, although no significant relationship was found with various other clinicopathological factors. There was no correlation of the TAAI with serum CA19-9 and CEA (Supplementary Table S4). Moreover, multivariate survival analysis revealed that the TAAI and these serum biomarkers were independent prognosticators (Supplementary Tables S2 and S3).

**Table 4:** Relationship between clinicopathological characteristics and tissue amino index (pancreatic ductal adenocarcinoma cohort 1).

Characteristics		No. of patients	Tissue amino index		*P*
			Low	High	
Age, years					0.442
	<60	93	45	48	
	≥60	37	15	22	
Sex					**0.019**
	Male	78	29	49	
	Female	52	31	21	
Pathologic tumor status					0.152^§^
	T1a	0	0	0	
	T1b	0	0	0	
	T1c	7	4	3	
	T2	77	40	37	
	T3	46	16	30	
	T4	0	0	0	
Pathologic node status					0.894
	N0	32	15	17	
	N1	47	23	24	
	N2	51	22	29	
Pathologic metastasis status					0.595
	M0	114	54	60	
	M1	16	6	10	
Stage					0.510^§^
	IA	5	3	2	
	IB	20	11	9	
	IIA	7	1	6	
	IIB	47	23	24	
	III	36	16	20	
	IV	15	6	9	
Tumor histological grade					0.056^§^
	G1	10	7	3	
	G2	98	47	51	
	G3	22	6	16	
Tumor margin status					0.855
	Negative	84	38	46	
	Positive	46	22	24	
Nerve plexus invasion^*^					0.440
	Absence	38	20	18	
	Presence	92	40	52	
Lymphatic invasion^*^					0.849
	0, 1	38	17	21	
	2, 3	92	43	49	
Venous invasion^*^					1.000
	0, 1	28	13	15	
	2, 3	102	47	55	
Intrapancreatic neural invasion^*^					0.276
	0, 1	49	26	23	
	2, 3	81	34	47	
Adjuvant chemotherapy^†^					1.000
	Absence	44	20	24	
	Presence	78	35	43	
Total		130	60	70	

^*^Classified according to the classification of pancreatic carcinoma of Japan Pancreas Society.

^§^Comparisons of qualitative variables are performed using the χ^2^ test, and otherwise by Fisher’s exact test.

^†^Number of patients who we had information of chemotherapy was 122.

Amino acids in cancer tissues have been measured in several studies, although only a few reports have demonstrated that tissue amino acid profiles can serve as biomarkers for prognosis and cancer progression. One study showed that 15 metabolites, including Glu, Asp, a-ABA, and Cys, could predict the recurrence rate and survival for patients after surgery and chemotherapy^24^. The selected metabolites predicted outcomes in 4 cohorts of patients with colorectal cancer, although they did not predict outcomes in a cohort of patients with gastric cancer. Another group reported that the concentrations of 9 amino acids (Met, Val, Ile, Tyr, Pro, Phe, Leu, His, HyPro) were upregulated in various tissues in colorectal carcinogenesis^40^. Most of the amino acids whose concentrations altered in these tissues are essential or semi-essential amino acids, and suggested to be involved in cell transformation.

Certain amino acids have been reported to show a close correlation with malignant tumors^41^, although our study revealed that this correlation could not be consistently detected. Here, tissue amino acid profiles were similar between an indolent tumor SPN, and a highly aggressive tumor ANA. We suggest that further careful investigation is necessary for characterizing the amino acid profiles of each tumor or lesion.

A study comparing the plasma amino acid profiles of patients with PDAC and healthy controls suggested that these profiles could be used for assessing PDAC risk^19^. However, the relationship between the amino acid profiles of plasma and each selected tissue needs to be determined. Similarly, the mechanism that might underlie the development of these profiles remains to be elucidated.

The limitations of our study are that this was mainly a retrospective analysis, the small cohorts and a slight delay during blood-supply stoppage and tissue collection. Although the tissue samples collection and freezing was performed quickly, the possibility of the altered amino acid profiles due to small amount of metabolite degradation during blood supply stoppage and freezing cannot be excluded.

In conclusion, we analyzed tissue amino acid profiles and showed that 1) tissue amino acid profiles are characteristic of pancreatic tumor types and lesions; 2) tissue components are directly related to amino acid concentration; 3) some of the profiles are closely associated with PDAC carcinogenesis and cancer progression; and 4) TAAI could serve as an independent prognosticator for patients with PDAC. Several types of cancer show elevated tissue amino acid levels relative to normal tissue counterpart as the proliferation and glycolysis-related metabolites are enriched in tumors. Moreover, amino acid concentrations increase due to the increased catabolism in tumor tissues. In contrast, not all cancer tissues exhibit an increase in every amino acid, as shown in PDAC. With absolute values for evaluating tissue amino acid profiles, we can compare the profiles of distinct organs, tissues, or species. When coupled with the findings of further validation studies conducting by other research groups using other cohorts, these characteristic tissue amino acid profiles could potentially be used as pancreatic tumor biomarkers in clinical diagnosis.

## Methods

### Study population

We first selected 323 patients with pancreatic tumors or lesions who had undergone initial surgical resection between 2004 and 2011 at the National Cancer Center Hospital, and obtained fresh frozen tissues from the resected surgical specimens. Only those fresh frozen tissues in which the tumor tissue or lesion occupied >90% of the total tissue area in cryostat sections were used from 240 patients (Table 1). Particularly, in the case of noninvasive intraductal papillary-mucinous neoplasm (IPMN), the surrounding non-tumorous tissue frequently occupied >10% of the total tissue area; hence, we used only 26 of the initial 80 cases. Normal tissues were obtained from 18 patients with non-pancreatic tumors. None of the patients had received any therapy before surgery. All patients included in this study had undergone macroscopic curative resection. The clinicopathological characteristics of the PDAC patients are summarized in Table 4. Survival analysis was performed on conventional PDAC cases; anaplastic carcinoma (ANA), invasive carcinoma associated with IPMN (IPMC-IC) and mucinous cystic neoplasm (MCN) were excluded. The median follow-up periods post-surgery for all included patients and living patients were 21.1 (3.1–88) and 27.4 (6.0–88) months, respectively. At the census date (September 2011), 47 patients (36.2%) were alive, 74 (56.9%) had died of pancreatic cancer, and 9 (6.9%) had died of other causes. All M1^42^ patients exhibited nodal metastasis around the abdominal aorta without any other form of metastasis.

For validation, we collected samples prospectively from January 2013 to July 2015: Cohort 2 comprised 98 patients who had undergone initial surgical resection for PDAC at the National Cancer Center Hospital, Tokyo, and their fresh frozen tissues were obtained from the resected surgical specimen. The fresh frozen tissues were used only if the tumor tissue occupied >90% of the tissue area in cryostat sections. Thus, 71/98 PDAC patients were analyzed (demographic information in Supplementary Table S4).

### Pathological examination and immunohistochemistry

All tumors were examined pathologically and classified according to the World Health Organization (WHO) classification^20^, UICC TNM classification^42^, and the Japanese Pancreas Society classification of pancreatic carcinoma^43^.

Immunohistochemistry was performed on cryostat sections as described previously^44^. We used antibodies against the following: Chromogranin A (1:100), CD45 Leucocyte common antigen (1:100), CD68 (1:250), epithelial membrane antigen (EMA) (1:200) and cytokeratins AE1/AE3 (1:200) from DAKO (Glostrup, Denmark), and Bcl-10 (331.3; 1:100) from Santa Cruz Biotechnology (Santa Cruz, CA). Immunohistochemistry without the primary antibody was considered as negative control. Aniline blue staining and Sudan-III staining (Muto pure chemicals, Tokyo, Japan) were performed as instructed. After immunohistochemistry or staining, the microscopic images were imported as digital photo files using a NanoZoomer Digital Pathology system (Hamamatsu Photonics, Hamamatsu, Japan), and the density of the immunolabeled cells or stained area was analyzed using the image analysis software, Tissue Studio (Definiens, Munich, Germany).

### Measurement of tissue amino acids

The procedure for measuring tissue amino acids is summarized in Fig. 1a. In IPMC-IC cases, we analyzed the invasive cancer-lesion tissue. The procedure details have been previously described^17^. Briefly, fresh tissues were obtained after surgical treatment and the excised tissues were cut into 1.0 cm^3^ pieces, immediately frozen in Tissue-Tek OCT compound (Sakura Fineteck Japan, Tokyo, Japan). The frozen blocks of pancreatic tissues were sliced into 12 serial sections in 6 µm thick by using a cryostat. Images of the first and the twelfth sections were captured by a scanner NanoZoomer and the average tissue area was measured using Image-J software. The weights of the remaining 10 sections were calculated by multiplying the average area and thickness of each sample with a specific gravity of 1.0. All 10 sections in 1 mL of 80% methanol containing 6 µM phenyl-*d*_*5*_-alanine were homogenated. After centrifuging, the supernatants were extracted in chloroform, and the aqueous phase was dried and dissolved in the purified water. After adding AccQ Fluor borate buffer and AccQ Fluor reagent solution, the mixture was heated. After cooling, 0.2% acetic acid solution was added to the mixture. LC-MS/MS analysis was performed on a Shimadzu Nexera MP system equipped with an LCMS-8030PLUS mass spectrometer (Shimadzu, Kyoto, Japan). Data was acquired and processed using LC-MS solutions software (version 5.60 SP1). Analytical conditions are described elsewhere^17^.

### Statistical analysis

Individual tissue amino acid concentrations were normalized relative to total amino acid concentrations. Differences in normalized values, i.e. amino acid concentration ratios, among tissue types were analyzed using Steel’s multiple-comparison test or Dunn’s multiple-comparison test after Kruskal-Wallis test.

To evaluate the similarity of amino acid patterns of each type—normal pancreas (N), chronic pancreatitis (CP), IPMN, IPMC-IC, PDAC, ANA, ACC, NET and SPN—hierarchical clustering (Ward’s minimum variance method) was performed by using the median of amino acid concentration ratios of each tissue type. Distinct amino acid patterns that contribute to disease classification were extracted using principal component analysis (PCA). In these analyses, data was first standardized by calculating the z-scores.

To examine the direct relation between amino acid concentration and tissue occupancy of each tissue component (*e.g*. fibrous tissue, fat tissue, acinar cells, etc.), path analysis (multivariate regression analysis) was performed (BellCurve for Excel, Social Survey Research Information, Tokyo, Japan).

To examine the amino acid concentration trends during IPMN-associated progression IPMA, IPMC, IPMC-IC, PDAC and PDAC (common type) with ANA, we conducted the Jonckheere-Terpstra trend test followed by Bonferroni test. For TAAI estimation, the Cox proportional hazards model with variable selection was implemented by using the tissue amino acid profiles of patients with PDAC as explanatory variables. Stepwise variable selection performed to minimize Akaike Information Criterion (AIC).

According to the obtained model, the TAAI score of the *i*-th subject was calculated as

$$TAAIscor{e}_{j}=\sum _{i,j}{\beta }_{j}{x}_{ij}$$

where β_*j*_ was the estimated coefficient of the *j*-th amino acid, and x_*ij*_ was the standardized concentration of the *j*-th amino acid of the *i*-th subject. Cutoff levels for Kaplan-Meier analysis between high and low TAAI values were determined from the receiver operating characteristic (ROC) curve.

JMP^®^ 10 (SAS Institute Inc., Cary, NC) was used for Steel’s multiple-comparison test and cluster analysis. GraphPad Prism (GraphPad Software, La Jolla, CA) was used for Dunn’s comparison test. All the other statistical process was performed using the R language. For principal component analysis, “princomp” function, for Jonckheere-Terpstra trend test, “JonckheereTerpstraTest” function in the “DescTools” package, for estimation of the Cox proportional hazards model, Kaplan-Meier analysis and log-rank test, functions “coxph”, “survfit”, and “survdiff” in the “survival” package, for stepwise variable selection, “step” function in the “MASS” package, and for ROC analysis, “roc” function in the “pROC” package were used, respectively. *P* < 0.05 was considered statistically significant.

### Ethical approval and informed consent

The National Cancer Center Institutional Review Board approved this study (#2009-158, #2012-063). Informed consent was obtained from all participants involved in this study and all clinical investigation was conducted according to the principles expressed in the Declaration of Helsinki.

## Supplementary information

Supplementary information

## Data Availability

The datasets used and analyzed during the current study are available from the corresponding author upon reasonable request.
