# Supplementary material for: Tissue amino acid profiles are characteristic of tumor type, malignant phenotype, and tumor progression in pancreatic tumors
Source: Sci Rep. 2019 Jul 8;9:9816. doi: 10.1038/s41598-019-46404-4 (PMC6614459; doi:10.1038/s41598-019-46404-4)
Supplement: Supplementary file 1 — Supplementary information [file 41598_2019_46404_MOESM1_ESM.pdf]

Tissue amino acid profiles are characteristic of tumor type, malignant phenotype, and tumor progression in pancreatic tumors

Nobuyoshi Hiraoka*,[1,2] Sakino Toue,[4] Chisato Okamoto[5], Shinya Kikuchi[4], Yoshinori Ino[1], Rie Yamazaki-Itoh[1], Minoru Esaki[3], Satoshi Nara[3], Yoji Kishi[3], Akira Imaizumi[5], Nobukazu Ono[5], Kazuaki Shimada[3]

[1]Division of Molecular Pathology, National Cancer Center Research Institute, Tokyo,

[2]Division of Pathology and Clinical Laboratories and [3]Hepato-Biliary and Pancreatic Surgery Division, National Cancer Center Hospital, Tokyo

[4]Research Institute for Bioscience Products and Fine Chemicals, and [5]Institute for Innovation, Ajinomoto Co., Inc., Kanagawa

*correspondence, E-mail: nhiraoka@ncc.go.jp

Supplementary Table S1. Median (min to max) tissue amino acid concentrations used in this study

	N (n=18)	CP (n=15)	IPMN (n=26)	IPMC-IC (n=18)	PDAC (n=130)	ANA (n=6)	ACC (n=10)	NET (n=25)	SPN (n=10)
Gly	4368.2(2246.7 to 7270.0)	1843.6(1126.9 to 2774.1)	1755.6(898.1 to 6259.1)	2278.0(650.2 to 6018.8)	1998.7(679.8 to 7018.0)	2512.2(2122.3 to 4159.4)	4126.6(1941.9 to 6982.8)	1733.7(431.8 to 4515.7)	2166.7(937.6 to 3328.5)
Ala	5657.7(2949.1 to 9174.8)	1334.3(477.7 to 3361.6)	1986.2(775.7 to 4426.2)	1726.3(818.4 to 5961.6)	1694.6(495.8 to 4313.4)	2081.1(1224.4 to 4564.5)	3702.9(2700.2 to 7861.9)	1282.6(386.0 to 2991.9)	1963.3(620.2 to 3200.3)
GABA	170.4(52.0 to 296.8)	181.3(40.9 to 243.4)	35.3(19.8 to 178.2)	46.7(20.7 to 197.4)	66.0(14.1 to 304.0)	22.2(10.2 to 78.2)	39.5(13.5 to 204.4)	221.2(13.0 to 2343.4)	30.3(7.9 to 45.5)
a-ABA	97.7(38.5 to 202.1)	49.8(28.6 to 89.4)	53.5(27.6 to 74.6)	39.4(31.6 to 51.1)	55.4(20.2 to 180.4)	48.1(31.4 to 53.8)	58.6(35.7 to 126.7)	37.3(13.7 to 113.0)	89.4(39.8 to 184.3)
Ser	991.3(386.8 to 1606.7)	308.0(217.0 to 506.7)	441.0(188.7 to 960.3)	497.4(223.7 to 1264.6)	458.7(202.7 to 4525.8)	711.6(609.1 to 822.3)	787.3(551.1 to 1667.0)	332.7(74.4 to 916.1)	895.9(403.0 to 1575.3)
Pro	924.4(536.1 to 1937.2)	490.3(166.6 to 707.1)	536.3(222.1 to 1867.9)	647.0(263.1 to 1112.0)	681.6(269.3 to 1844.9)	1106.0(871.2 to 1566.1)	1336.7(627.6 to 1852.1)	371.6(128.2 to 960.6)	853.5(356.5 to 2341.9)
Val	425.9(232.9 to 857.0)	341.6(205.8 to 469.4)	409.8(167.7 to 1236.9)	408.6(158.5 to 711.3)	451.0(162.1 to 1010.4)	862.1(697.8 to 1242.1)	587.2(315.9 to 1350.3)	318.7(109.3 to 609.7)	648.1(230.6 to 1294.1)
Thr	937.9(442.5 to 1862.4)	352.4(218.5 to 607.7)	347.5(135.2 to 837.1)	451.0(204.9 to 1186.3)	460.6(175.3 to 1066.7)	778.6(625.5 to 1079.9)	730.4(610.5 to 1723.6)	260.4(70.3 to 895.9)	765.2(373.8 to 1177.8)
Tau	2483.1(1331.2 to 12848.9)	7215.4(4070.2 to 13514.7)	8963.4(1834.0 to 15485.6)	7573.6(1238.1 to 13353.4)	8533.9(2905.4 to 14567.3)	5278.1(2080.9 to 9403.9)	5978.9(1068.7 to 16067.9)	7855.6(1094.3 to 28385.0)	1748.6(354.5 to 4009.5)
HyPro	47.8(25.6 to 123.5)	50.0(19.2 to 81.2)	19.8(12.2 to 27.9)	32.4(1.5 to 59.6)	53.2(1.8 to 233.9)	37.4(21.0 to 81.3)	41.1(19.0 to 78.8)	18.6(2.9 to 46.0)	29.2(11.3 to 38.6)
Ile	207.7(96.5 to 458.9)	151.6(75.3 to 248.8)	199.8(77.6 to 605.5)	182.5(72.6 to 424.7)	222.5(84.2 to 453.7)	381.5(310.3 to 560.8)	318.5(127.3 to 692.8)	130.4(43.1 to 227.5)	211.9(64.4 to 448.4)
Leu	402.9(212.3 to 1186.0)	334.5(167.6 to 495.9)	395.3(172.6 to 1837.6)	377.1(118.6 to 820.2)	419.6(195.9 to 1040.1)	1001.2(798.6 to 1242.8)	725.1(294.2 to 1670.9)	280.8(103.0 to 595.5)	512.4(141.8 to 981.0)
Asn	520.4(243.4 to 963.0)	176.1(88.2 to 282.9)	176.1(75.7 to 377.7)	203.0(51.9 to 437.5)	216.3(80.3 to 603.2)	412.3(367.3 to 522.6)	445.7(256.9 to 720.4)	131.8(45.2 to 340.7)	298.8(200.0 to 444.8)
Orn	227.3(100.9 to 393.6)	76.4(35.1 to 238.5)	57.0(12.3 to 155.5)	69.2(34.7 to 197.2)	64.4(14.6 to 1377.1)	175.6(43.1 to 383.8)	212.3(102.2 to 551.2)	99.4(17.6 to 226.7)	55.5(37.7 to 150.9)
Asp	1503.1(561.9 to 3180.6)	1003.5(561.7 to 1386.9)	803.6(177.9 to 4358.1)	616.6(177.4 to 1475.2)	647.9(114.2 to 2595.6)	602.1(301.1 to 1123.8)	791.7(394.3 to 2074.6)	1767.6(148.8 to 7648.8)	285.8(97.1 to 805.1)
Gln	3241.4(1141.3 to 6820.4)	1036.6(690.3 to 1753.8)	1134.7(468.9 to 3221.0)	1090.7(235.8 to 3781.7)	1175.7(356.2 to 4838.4)	1703.1(988.1 to 2730.6)	3053.0(986.9 to 3731.0)	531.9(16.5 to 3472.0)	3319.4(1074.3 to 4431.2)
Lys	685.1(387.5 to 1668.9)	436.0(215.4 to 667.2)	475.2(160.3 to 1554.2)	433.6(99.9 to 1043.5)	412.1(118.9 to 975.3)	825.8(472.2 to 980.0)	779.5(477.3 to 1877.9)	581.3(218.3 to 1441.2)	365.8(220.5 to 585.2)
Glu	5383.4(2605.6 to 9223.9)	3714.1(1816.0 to 6245.7)	2209.8(1208.8 to 4404.5)	3138.2(1227.8 to 13020.5)	3543.6(1177.7 to 9828.9)	3185.7(2268.5 to 5408.1)	4711.8(2204.0 to 7553.9)	3237.6(674.2 to 6358.0)	2733.4(987.8 to 4297.6)
Met	38.6(10.2 to 201.6)	56.3(39.4 to 107.4)	79.9(35.4 to 242.4)	82.5(36.1 to 180.0)	93.2(37.7 to 227.3)	172.4(141.8 to 223.7)	83.5(31.1 to 380.7)	54.6(21.3 to 130.7)	148.7(61.8 to 333.7)
His	476.2(166.0 to 807.1)	150.1(104.9 to 255.5)	142.3(59.7 to 403.7)	148.4(96.0 to 350.7)	168.5(75.8 to 828.7)	332.2(233.8 to 496.0)	369.4(251.6 to 671.6)	131.6(51.2 to 1812.4)	334.2(112.7 to 516.7)
HyLys	1.9(0.3 to 7.4)	4.8(0.5 to 25.2)	35.7(8.5 to 144.3)	3.2(0.5 to 22.0)	4.7(0.4 to 27.0)	12.2(4.1 to 131.2)	18.7(10.5 to 42.1)	22.0(2.3 to 68.1)	13.3(10.8 to 26.7)
Phe	192.0(99.1 to 552.9)	146.1(70.0 to 201.0)	178.4(84.1 to 798.2)	181.6(69.1 to 389.4)	204.8(78.9 to 503.2)	446.3(359.9 to 578.6)	302.4(157.2 to 761.4)	130.7(48.4 to 293.5)	355.3(126.1 to 712.9)
Arg	137.3(55.0 to 730.6)	195.3(122.6 to 289.8)	274.7(109.5 to 1372.2)	228.9(116.9 to 503.1)	241.7(73.1 to 617.5)	306.4(208.1 to 435.4)	209.7(119.3 to 389.5)	212.6(25.3 to 909.9)	170.0(106.0 to 286.3)
Cit	30.6(9.7 to 62.2)	31.5(14.3 to 56.8)	24.5(4.6 to 57.6)	51.5(19.4 to 124.0)	44.5(6.4 to 147.3)	50.9(24.0 to 79.6)	31.8(8.7 to 412.5)	15.8(2.9 to 77.7)	79.7(41.3 to 175.4)
Tyr	139.5(87.2 to 507.1)	130.9(69.6 to 199.9)	162.5(62.1 to 780.4)	210.9(82.2 to 426.7)	205.0(88.1 to 529.2)	392.6(317.2 to 485.2)	279.7(130.8 to 659.0)	105.6(43.7 to 310.4)	490.4(187.2 to 890.4)
Trp	64.5(33.0 to 154.9)	57.3(28.1 to 82.4)	67.0(28.1 to 215.2)	71.3(35.7 to 125.0)	73.2(37.3 to 183.2)	137.7(106.5 to 200.2)	98.0(53.8 to 244.2)	48.3(16.9 to 148.2)	127.9(52.2 to 207.6)

N: normal pancreas, CP: chronic pancreatitis, IPMN: intraductal papillary-mucinous neoplasm, IPMC-IC: IPMN associated with invasive carcinoma, PDAC: pancreatic ductal adenocarcinoma, ANA: anaplastic carcinoma, ACC: acinar cell carcinoma, NET: neuroendocrine neoplasm, SPN: solid-pseudopapillary neoplasm

Supplementary Table S2. Univariate and multivariate analysis of prognostic factors associated with overall survival in patients with PDAC (cohort 2) (n=71)

	Univariate analysis		Multivariate analysis	
Variables	HR (95% CI)	*P* value*	HR (95% CI)	*P* value
Age (<60/ ≥60 years)	2.757 (1.177-6.455)	**0.020**		
Gender (male/ female)	1.073 (0.463-2.487)	0.870		
Pathologic tumor status (T2+T3/ T1)	4.621 (0.621-34.399)	0.135		
Pathologic node status (N1+N2/ N0)	4.531 (1.339-15.332)	**0.015**	4.402 (1.250-15.506)	**0.021**
Pathologic metastasis status (M1/ M0)**	1.783 (0.239-13.310)	0.573		
Histological grade (G2 + G3/ G1)	1.731 (0.233-12.878)	0.592		
Tumor margin status (positive/ negative)	1.437 (0.602-3.427)	0.414		
Lymphatic invasion (2,3/ 0,1)	9.779 (1.314-72.794)	**0.026**	8.922 (1.167-68.182)	**0.035**
Venous invasion (2,3/ 0,1)	4.089 (0.549-30.446)	0.169		
Intrapancreatic neural invasion (2,3/ 0,1)	4.960 (1.157-21.267)	**0.031**		
Nerve plexus invasion (positive/ negative)	3.399 (1.253-9.222)	**0.016**		
Chemotherapy (negative/ positive)	2.707 (1.153-6.356)	**0.022**		
serum CA19-9 (>37 U/ml/ ≤37 U/ml)	5.969 (1.394-25.559)	**0.016**		
serum CEA (>5 ng/ml/ ≤5 ng/ml)	2.550 (1.036-6.277)	**0.042**	3.164 (1.028-9.737)	**0.045**
Tissue amino acid index (high/ low)	2.639 (1.018-6.842)	**0.046**	3.000 (1.135-7.927)	**0.027**

Supplementary Table S3. Univariate and multivariate analysis of prognostic factors associated with disease-free survival in patients with PDAC (cohort 2) (n=71)

Age (<60/ ≥60 years)	2.155 (1.227-3.785)	**0.008**		
Gender (male/ female)	1.095 (0.650-1.845)	0.732		
Pathologic tumor status (T2+T3/ T1)	7.328 (1.781-30.143)	**0.006**		
Pathologic node status (N1+N2/ N0)	2.504 (1.366-4.591)	**0.003**	2.074 (1.085-3.963)	**0.027**
Pathologic metastasis status (M1/ M0)**	2.402 (0.738-7.816)	0.146		
Histological grade (G2 + G3/ G1)	0.979 (0.391-2.455)	0.965		
Tumor margin status (positive/ negative)	1.030 (0.583-1.818)	0.919		
Lymphatic invasion (2,3/ 0,1)	4.566 (1.948-10.699)	**0.0005**	3.306 (1.376-7.942)	**0.008**
Venous invasion (2,3/ 0,1)	3.197 (1.153-8.865)	**0.026**		
Intrapancreatic neural invasion (2,3/ 0,1)	2.839 (1.348-5.821)	**0.004**		
Nerve plexus invasion (positive/ negative)	2.009 (1.156-3.492)	**0.013**		
Chemotherapy (negative/ positive)	1.609 (0.919-2.815)	0.096		
serum CA19-9 (>37 U/ml/ ≤37 U/ml)	2.843 (1.485-5.444)	**0.002**		
serum CEA (>5 ng/ml/ ≤5 ng/ml)	1.942 (1.035-3.641)	**0.039**		
Tissue amino acid profile (high/ low)	2.416 (1.304-4.476)	**0.005**	2.584 (1.384-4.824)	**0.003**

Supplemenary Table S4. Relationship between clinicopathological characteristics and tissue amino index (pancreatic ductal adenocarcinoma cohort 2)

		Tissue amino index		
Characteristics	No. of patients	Low	High	*P*
Age, years				0.781
<60	55	27	28	
≥60	16	7	9	
Sex				0.500
Male	37	16	21	
Female	34	18	16	
Pathologic tumor status				0.171§
T1a	0	0	0	
T1b	0	0	0	
T1c	7	1	6	
T2	50	26	24	
T3	14	7	7	
T4	0	0	0	
Pathologic node status				0.721
N0	30	13	17	
N1	20	11	9	
N2	21	10	11	
Pathologic metastasis status				0.503
M0	68	32	36	
M1	3	2	1	
Stage				0.338§
IA	5	0	5	
IB	21	11	10	
IIA	4	2	2	
IIB	20	11	9	
III	18	8	10	
IV	3	2	1	
Tumor histological grade				0.166§
G1	6	2	4	
G2	47	20	27	
G3	18	12	6	
Tumor margin status				0.311
Negative	50	26	24	
Positive	21	8	13	
Nerve plexus invasion*				1.000
Absence	28	13	15	
Presence	43	21	22	
Lymphatic invasion*				0.586
0, 1	17	7	10	
2, 3	54	27	27	
Venous invasion*				1.000
0, 1	7	3	4	
2, 3	64	31	33	
Intrapancreatic neural invasion*				1.000
0, 1	13	6	7	
2, 3	58	28	30	
Adjuvant chemotherapy†				0.436
Absence	21	12	9	
Presence	50	22	28	
Serum CA19-9				
≤37 U/ml	21	7	14	0.127
>37 U/ml	50	27	23	
Serum CEA				
≤5 ng/ml	59	29	30	0.756
>5 ng/ml	12	5	7	
Total	71	34	37	

*Classified according to the classification of pancreatic carcinoma of Japan Pancreas Society

§Comparisons of qualitative variables are performed using the χ^2 test, and otherwise by Fisher's exact test.

Supplementary Figure legends

Supplementary Figure S1.

(A) Amino acid profiles of normal and diseased pancreatic tissues. Scatter plots of PCA scores for each tissue; left, components 1 and 2; right, components 1 and 3.

(B) Distributions of PCA scores for each tissue (left panel) and PCA loadings for each amino acid (right panel). PC1 discriminated ACC, SPN and NET from N and other tumors, PC2 discriminated ACC and N from others, and PC3 discriminated NET and IPMN from N and others. Tau and Asp were positive contributors and Pro, Ile, Leu and Met were negative for PC1. Tau and Met were positive contributors and Ala and Gly were negative for PC2. HyPro was a positive contributor and Lys and Asp were negative for PC3.

(C) Cutoff point of tissue amino acid index (TAAI) receiver operating characteristic (ROC) curve analysis. Arrows indicates cutoff point. Y- and x-axis indicate true positive fraction (TPF) and falsse positive fraction (FPF), respectively.

(D) Kaplan-Meier survival curves showing comparison of overall survival (left panel) and disease-free survival (right panel) between high (red) and low (blue) of tissue amino acid index (TAAI) groups in cohort 2. *P* values were obtained from log-rank test. The "x" and "+" represent censoring and failure, respectively.

Supplementary Figure S2. The direct relation between tissue amino acid concentration and tissue components

Path analysis (multiple regression analysis) is performed using N (n=7) and CP (n=10). The direct relationship between tissue components and amino acid concentration is represented by means of low diagram. R^2 (coefficient of determination) is adjusted r-squared that the model explains all the variability of the response data around its mean. Path coefficients (standard partial regression coefficients) estimate the strength of the relationship between two variables. Tissue occupancy of each tissue component [Acn: Bcl-10 (331.3)$^+$ acinar cells, Islet: Chromogranin A (CGA)$^+$ islet cells, Duct: EMA$^+$ or Cytokeratin (CK, AE1/AE3)$^+$/Bcl-10$^-$/CGA$^-$ ductal epithelial cells in non-cancerous tissue, Mac: CD45$^+$/CD68$^+$ macrophages, Lym: CD45$^+$/CD68$^-$ lymphocytes, PDAC: EMA$^+$ or CK$^+$/Bcl-10$^-$/CGA$^-$ cancer cells, Fib: aniline blue$^+$ area, Fat: SudanIII$^+$ fat cells] is counted as the ratio of its area within the total area. Fib is omitted in calculation for inhibiting

multicollinearity. $^{\Delta}$, *, **, ***: $P < 0.10, 0.05, 0.01, 0.001$.

Supplementary Figure S3. Differences of amino acid concentration differences among normal and diseased pancreatic tissues. Upper panels, box plots of z-scores calculated from the values of amino acid concentration ratios in each tissue type. Lower panels, results of Dunn's multiple-comparison tests. *, **, ***, ****: $P < 0.05, 0.01, 0.001, 0.0001$.

Supplementary Figure S1

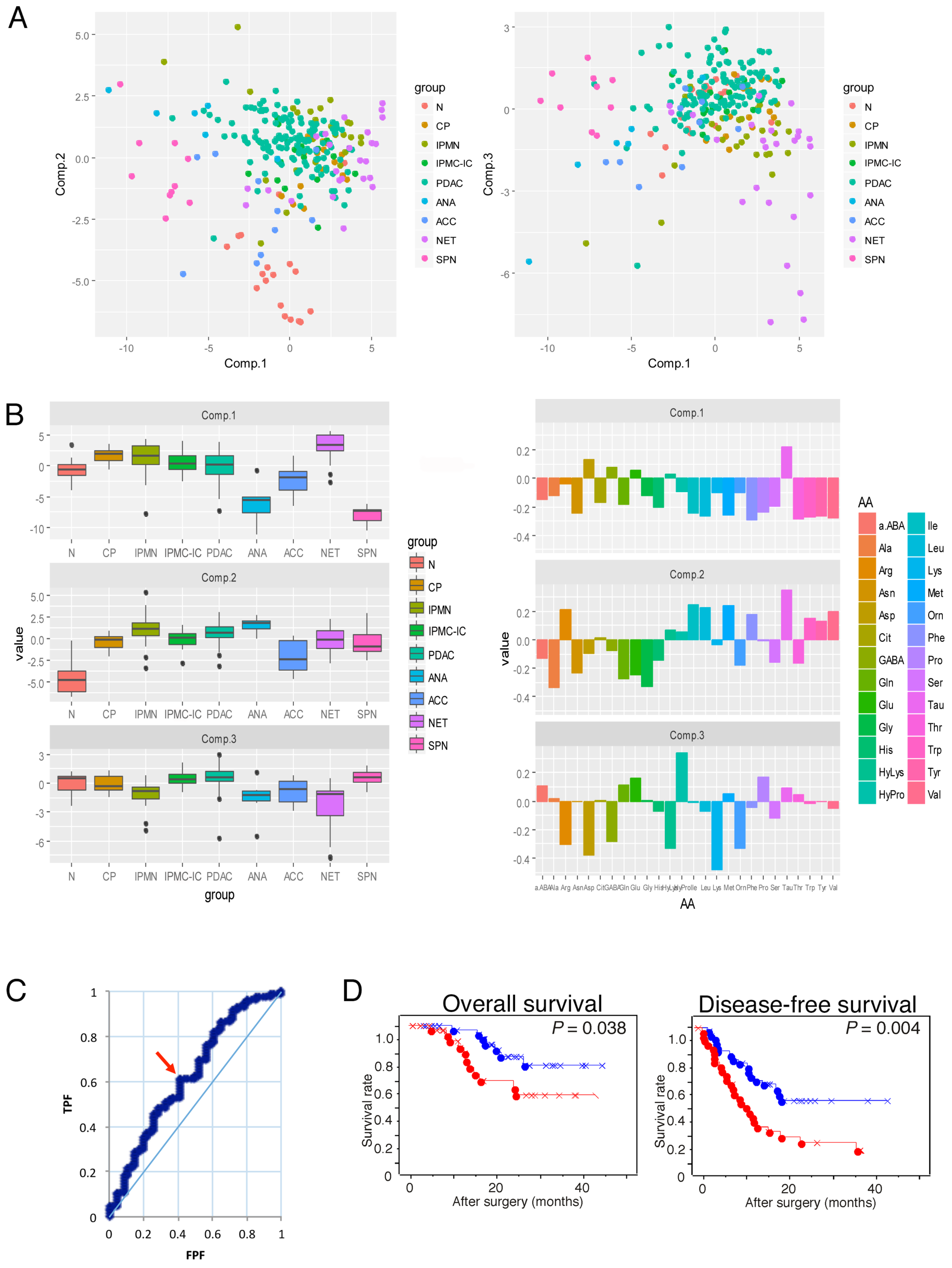

Supplementary Figure S2

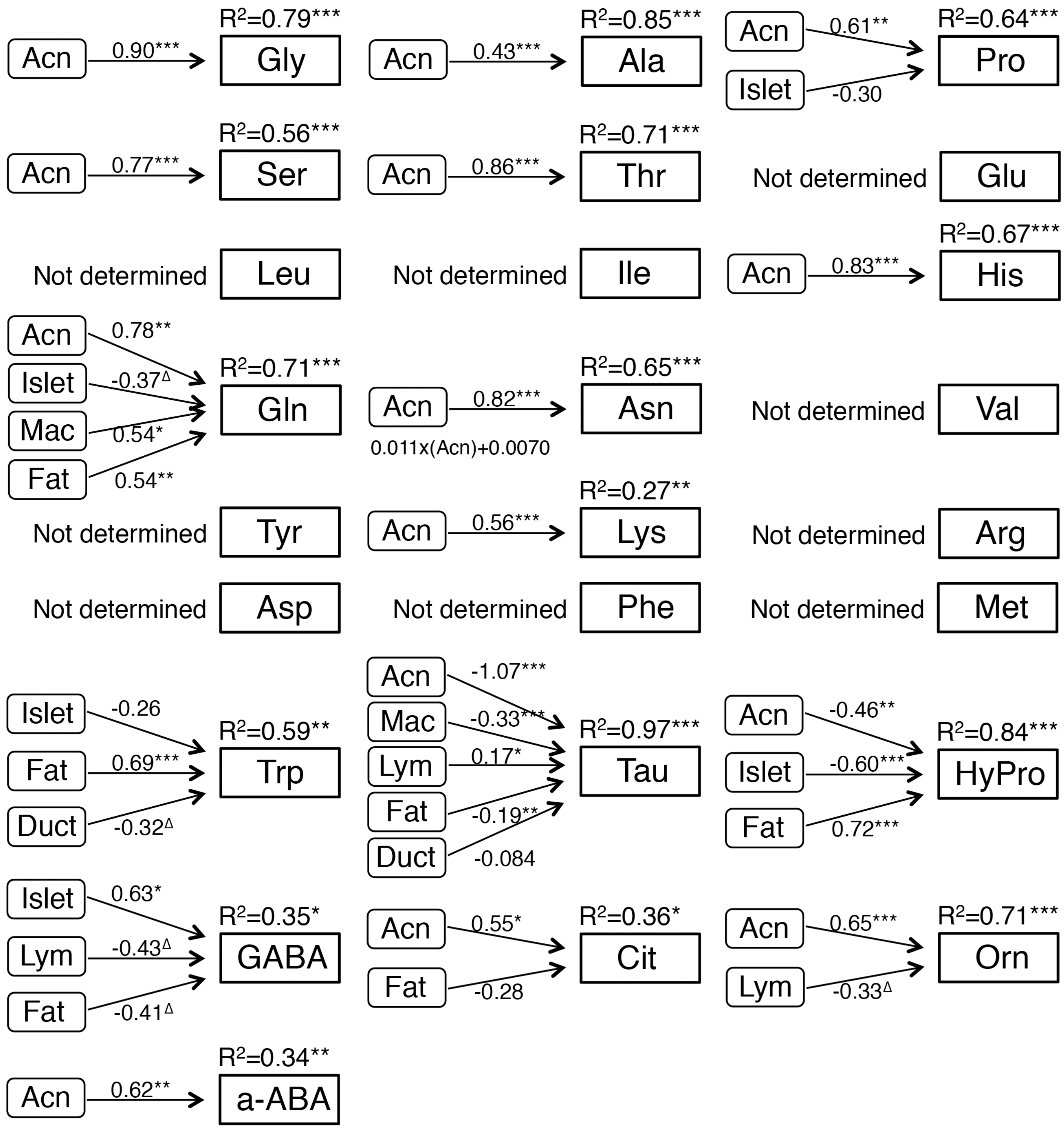

Supplementary Figure S3-(1)

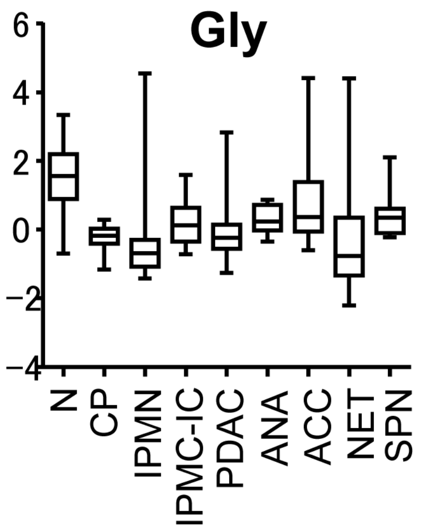

	N	CP	IPMN	IPMC-IC	PDAC	ANA	ACC	NET	SPN
N		**	****		****			****	
CP									
IPMN				***	*	*	***		***
IPMC-IC									
PDAC									
ANA									
ACC								*	
NET									*
SPN									

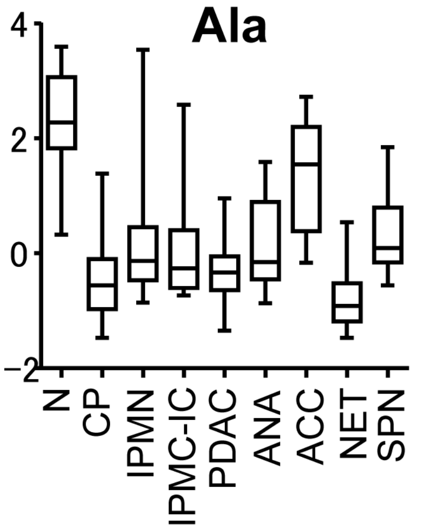

	N	CP	IPMN	IPMC-IC	PDAC	ANA	ACC	NET	SPN
N		****	**	**	****			****	
CP							***		
IPMN								***	
IPMC-IC								*	
PDAC							***	*	
ANA									
ACC								****	
NET									***
SPN									

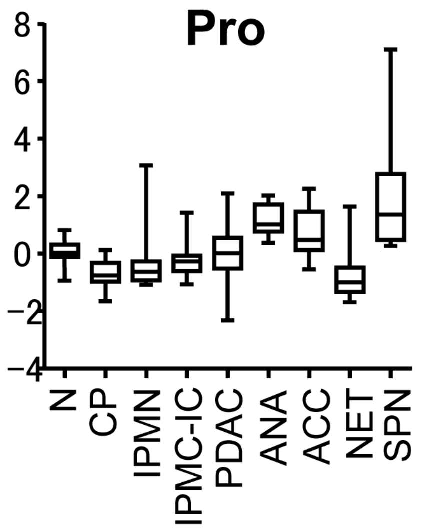

	N	CP	IPMN	IPMC-IC	PDAC	ANA	ACC	NET	SPN
N		*						**	
CP					**	***	**		****
IPMN					*	**	*		****
IPMC-IC						*			**
PDAC								****	*
ANA								****	
ACC								***	
NET									****
SPN									

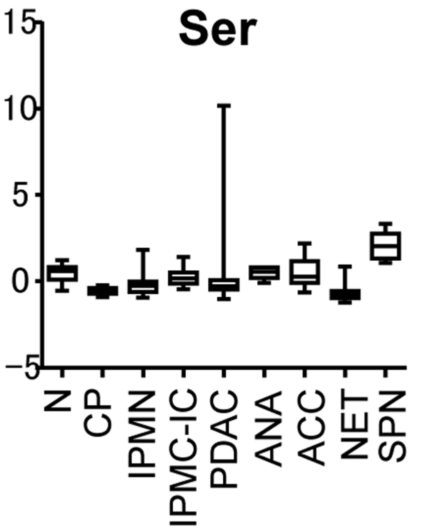

	N	CP	IPMN	IPMC-IC	PDAC	ANA	ACC	NET	SPN
N		****	**		***			****	
CP				***		**	**		****
IPMN									****
IPMC-IC								****	
PDAC								***	****
ANA								***	
ACC								****	
NET									****
SPN									

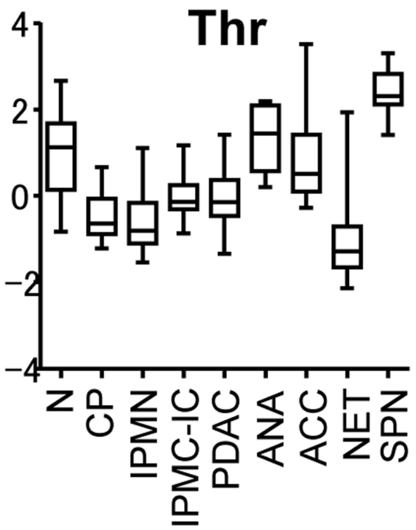

	N	CP	IPMN	IPMC-IC	PDAC	ANA	ACC	NET	SPN
N		***	****		**			****	
CP						**	*		****
IPMN					*	***	***		****
IPMC-IC								*	**
PDAC								****	****
ANA								****	
ACC								****	
NET									****
SPN									

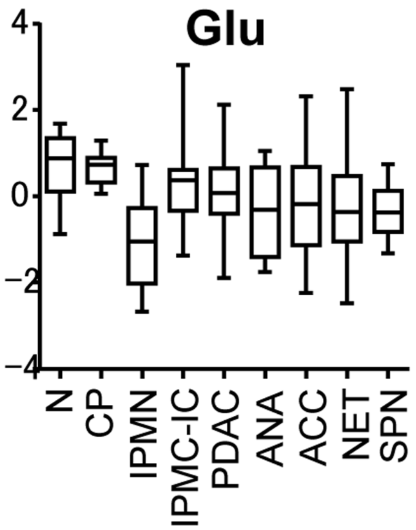

	N	CP	IPMN	IPMC-IC	PDAC	ANA	ACC	NET	SPN
N			****						
CP			****						
IPMN				**	****				
IPMC-IC									
PDAC									
ANA									
ACC									
NET									
SPN									

Supplementary Figure S3-(2)

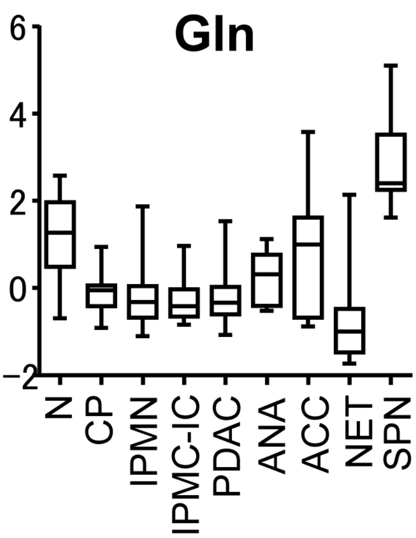

	N	CP	IPMN	IPMC-IC	PDAC	ANA	ACC	NET	SPN
N			**	**	****			****	
CP								*	*
IPMN									****
IPMC-IC									***
PDAC								*	****
ANA									
ACC								**	
NET									****
SPN									

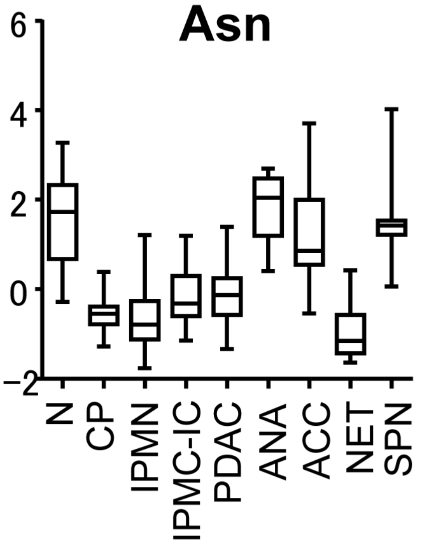

	N	CP	IPMN	IPMC-IC	PDAC	ANA	ACC	NET	SPN
N		****	****	***	****			***	
CP						**	**		***
IPMN					*	****	****		****
IPMC-IC						*			**
PDAC						*	*	****	**
ANA								****	
ACC								****	
NET									****
SPN									

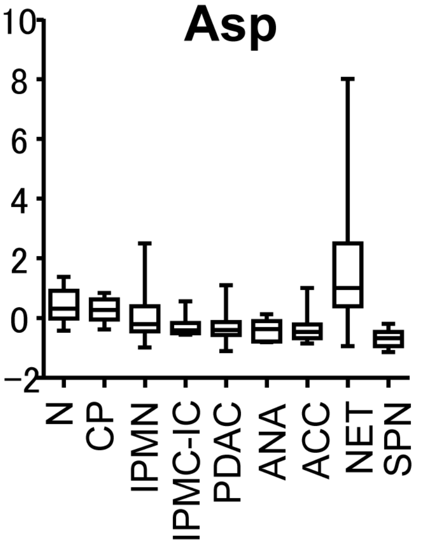

	N	CP	IPMN	IPMC-IC	PDAC	ANA	ACC	NET	SPN
N				*	****		*		****
CP					***		*		****
IPMN								*	*
IPMC-IC								***	
PDAC								****	
ANA								*	
ACC								***	
NET									****
SPN									

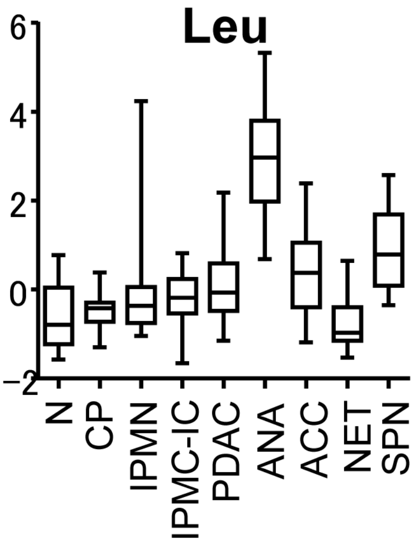

	N	CP	IPMN	IPMC-IC	PDAC	ANA	ACC	NET	SPN
N					*	***			**
CP						***			*
IPMN						**			
IPMC-IC						*			
PDAC						*		****	
ANA									
ACC								**	
NET									****
SPN									

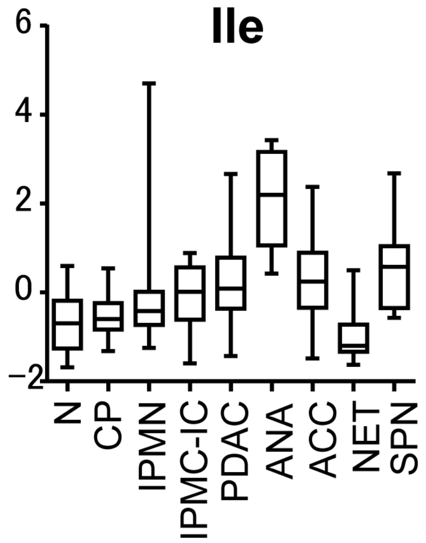

	N	CP	IPMN	IPMC-IC	PDAC	ANA	ACC	NET	SPN
N					**	***			*
CP					*	***			
IPMN						**			
IPMC-IC								**	
PDAC								****	
ANA								****	
ACC								**	
NET									****
SPN									

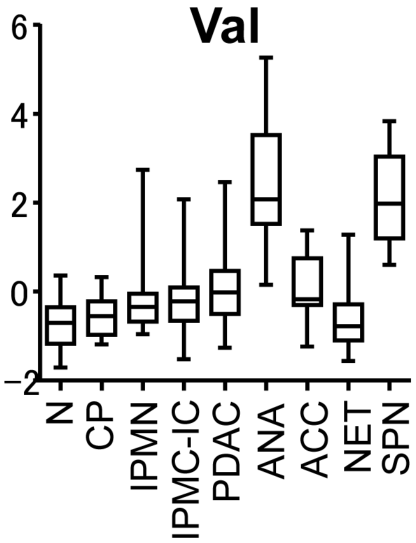

	N	CP	IPMN	IPMC-IC	PDAC	ANA	ACC	NET	SPN
N					**	****			****
CP						***			****
IPMN						*			***
IPMC-IC									**
PDAC								***	**
ANA								****	
ACC									
NET									****
SPN									

Supplementary Figure S3-(3)

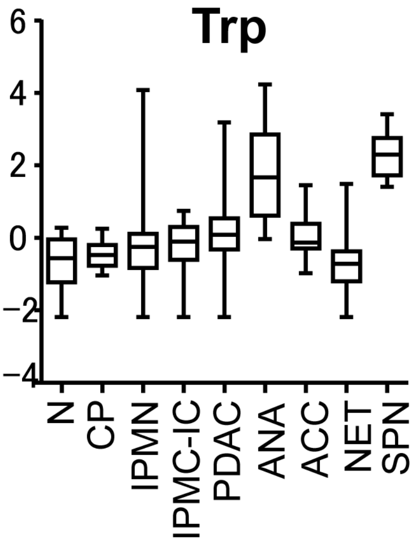

	N	CP	IPMN	IPMC-IC	PDAC	ANA	ACC	NET	SPN
N					**	***			****
CP						**			****
IPMN						*			****
IPMC-IC									***
PDAC								****	***
ANA								****	
ACC									*
NET									****
SPN									

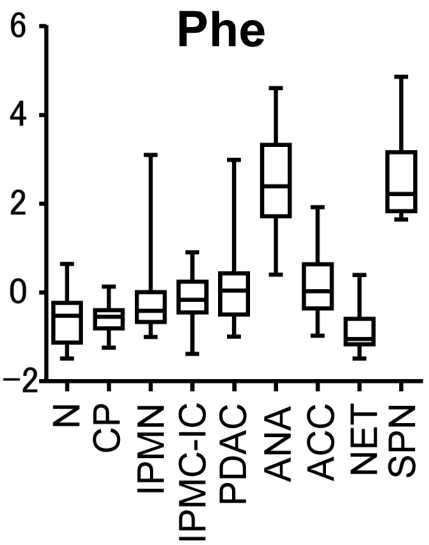

	N	CP	IPMN	IPMC-IC	PDAC	ANA	ACC	NET	SPN
N					*	***			****
CP					*	***			****
IPMN						**			****
IPMC-IC								**	**
PDAC								****	***
ANA								****	
ACC								**	
NET									****
SPN									

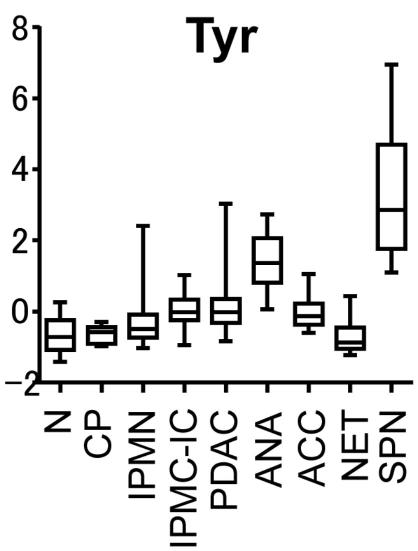

	N	CP	IPMN	IPMC-IC	PDAC	ANA	ACC	NET	SPN
N				*	***	****			****
CP				**	***	****			****
IPMN					*	**			****
IPMC-IC								***	
PDAC								****	**
ANA								****	
ACC									*
NET									****
SPN									

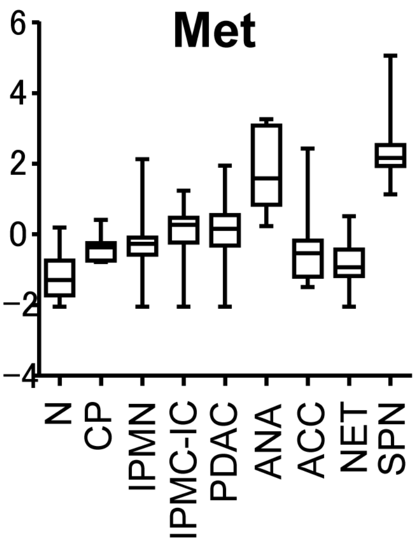

	N	CP	IPMN	IPMC-IC	PDAC	ANA	ACC	NET	SPN
N				***	****	****			****
CP						**			****
IPMN						**			****
IPMC-IC								**	*
PDAC								****	**
ANA							**	****	
ACC									****
NET									****
SPN									

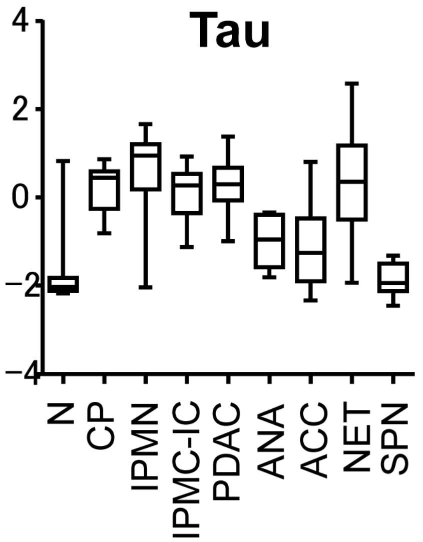

	N	CP	IPMN	IPMC-IC	PDAC	ANA	ACC	NET	SPN
N		***	****	**	****			****	
CP									**
IPMN						**	***		****
IPMC-IC									**
PDAC							**		****
ANA									
ACC								*	
NET									****
SPN									

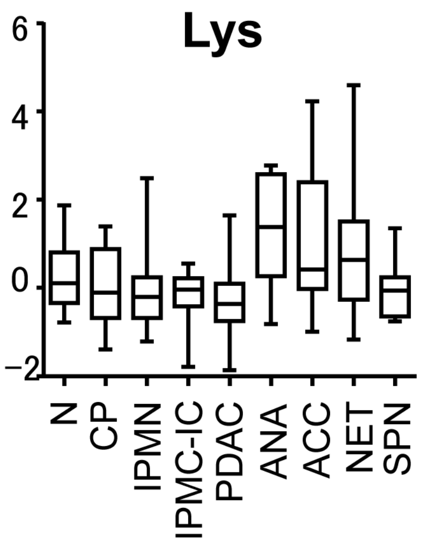

	N	CP	IPMN	IPMC-IC	PDAC	ANA	ACC	NET	SPN
N									
CP									
IPMN									
IPMC-IC									
PDAC								**	
ANA									
ACC									
NET									
SPN									

Supplementary Figure S3-(4)

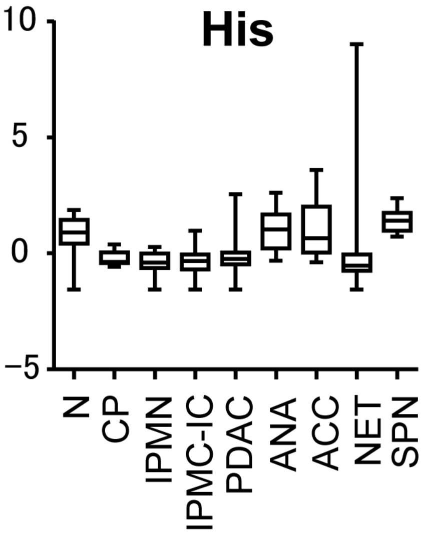

	N	CP	IPMN	IPMC-IC	PDAC	ANA	ACC	NET	SPN
N		*	****	***	****			****	
CP									**
IPMN						*	**		****
IPMC-IC							*		****
PDAC							*		****
ANA								*	
ACC								**	
NET									****
SPN									

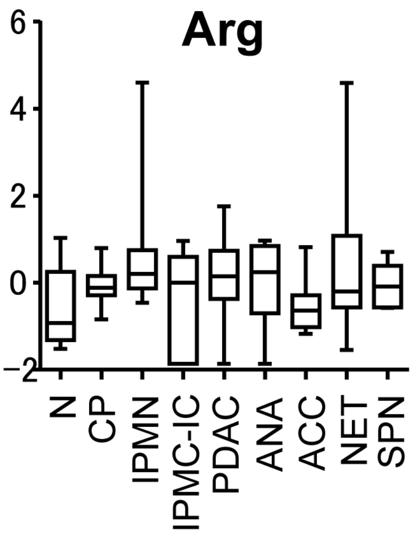

	N	CP	IPMN	IPMC-IC	PDAC	ANA	ACC	NET	SPN
N									
CP			*						
IPMN									
IPMC-IC									
PDAC									
ANA									
ACC									
NET									
SPN									

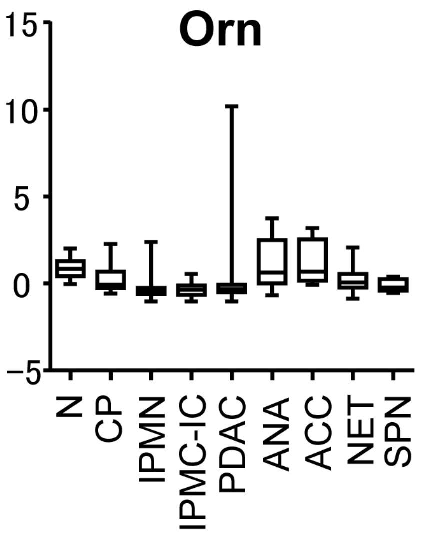

	N	CP	IPMN	IPMC-IC	PDAC	ANA	ACC	NET	SPN
N			****	****	****				
CP			*						
IPMN						*	****	**	
IPMC-IC							**		
PDAC							****	*	
ANA									
ACC									
NET									
SPN									

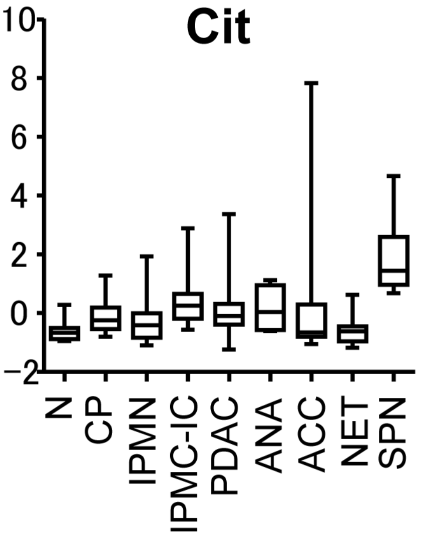

	N	CP	IPMN	IPMC-IC	PDAC	ANA	ACC	NET	SPN
N				***	**				****
CP									**
IPMN				*					****
IPMC-IC								****	
PDAC								***	**
ANA									
ACC									***
NET									****
SPN									

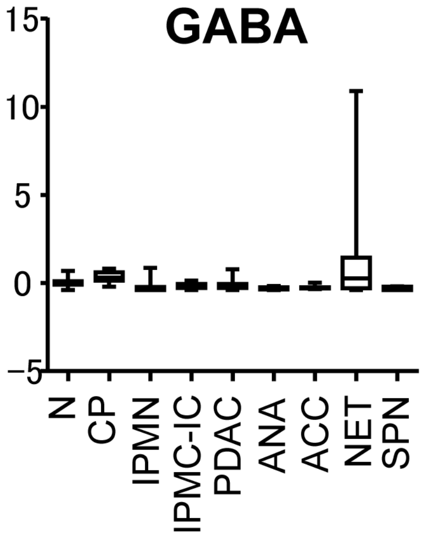

	N	CP	IPMN	IPMC-IC	PDAC	ANA	ACC	NET	SPN
N			****		*	*	*		***
CP			****	**	****	***	***		****
IPMN								***	
IPMC-IC									
PDAC									
ANA									
ACC									
NET									**
SPN									

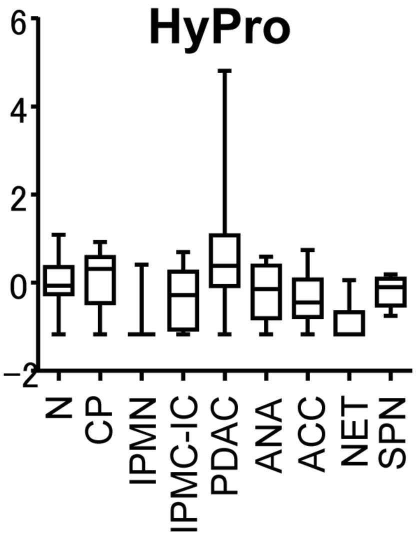

	N	CP	IPMN	IPMC-IC	PDAC	ANA	ACC	NET	SPN
N			**					*	
CP			**					*	
IPMN					****				
IPMC-IC									
PDAC								****	
ANA									
ACC									
NET									
SPN									

Supplementary Figure S3-(5)

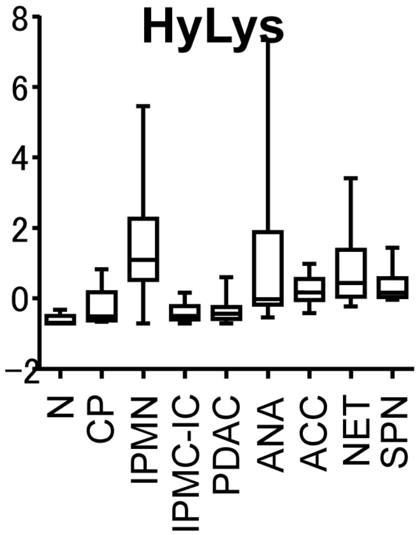

	N	CP	IPMN	IPMC-IC	PDAC	ANA	ACC	NET	SPN
N			****		*	**	****	****	****
CP			***					**	
IPMN				****	****				
IPMC-IC							*	****	*
PDAC							*	****	**
ANA									
ACC									
NET									
SPN									

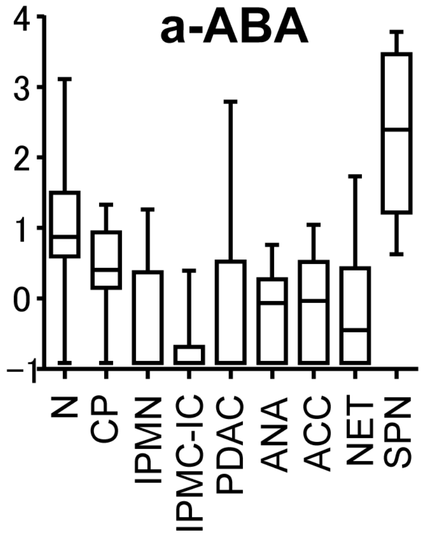

	N	CP	IPMN	IPMC-IC	PDAC	ANA	ACC	NET	SPN
N			***	****	***			**	
CP				**					
IPMN									****
IPMC-IC									****
PDAC									****
ANA									*
ACC									*
NET									****
SPN									
